# Differential alteration in *Lactiplantibacillus plantarum* subsp. *plantarum* quorum-sensing systems and reduced *Candida albicans* yeast survival and virulence gene expression in dual-species interaction

**DOI:** 10.1128/spectrum.00353-24

**Published:** 2024-05-08

**Authors:** Zhenbo Xu, Yaqin Li, Aijuan Xu, Liang Xue, Thanapop Soteyome, Lei Yuan, Qin Ma, Gamini Seneviratne, Wei Hong, Yuzhu Mao, Birthe V. Kjellerup, Junyan Liu

**Affiliations:** 1 Guangdong Province Key Laboratory for Green Processing of Natural Products and Product Safety, Engineering Research Center of Starch and Vegetable Protein Processing Ministry of Education, School of Food Science and Engineering, South China University of Technology, Guangzhou, China; 2 Department of Laboratory Medicine, the Second Affiliated Hospital of Shantou University Medical College, Shantou, Guangdong, China; 3 Guangzhou Hybribio Medical Laboratory, Guangzhou, China; 4 Guangdong Provincial Key Laboratory of Microbial Safety and Health, State Key Laboratory of Applied Microbiology Southern China, Institute of Microbiology, Guangdong Academy of Sciences, China, Guangzhou, Guangdong; 5 Key Laboratory of Agricultural Microbiomics and Precision Application, Ministry of Agriculture and Rural Affairs, Guangdong Academy of Sciences, Guangzhou, Guangdong, China; 6 Home Economics Technology, Rajamangala University of Technology Phra Nakhon, Bangkok, Thailand; 7 School of Food Science and Engineering, Yangzhou University, Yangzhou, Jiangsu, China; 8 Key Laboratory of Functional Foods, Ministry of Agriculture, Guangdong Key Laboratory of Agricultural Products Processing, Sericultural and Agri-Food Research Institute, Guangdong Academy of Agricultural Sciences, Guangzhou, China; 9 National Institute of Fundamental Studies, Kandy, Sri Lanka; 10 GMU-GIBH Joint School of Life Sciences, Guangzhou Medical University, Guangzhou, Guangdong, China; 11 Department of Civil and Environmental Engineering, University of Maryland, College Park, Maryland, USA; 12 Guangdong Provincial Key Laboratory of Lingnan Specialty Food Science and Technology, College of Light Industry and Food Science, Academy of Contemporary Agricultural Engineering Innovations, Zhongkai University of Agriculture and Engineering, Guangzhou, China; 13 Key Laboratory of Green Processing and Intelligent Manufacturing of Lingnan Specialty Food, Ministry of Agriculture, Guangzhou, China; South China Sea Institute of Oceanology Chinese Academy of Sciences, Guangzhou, Guangdong, China

**Keywords:** *Lactiplantibacillus plantarum *subsp. *plantarum*, *Candida albicans*, polymicrobial transcriptomics, quorum-sensing system, pathogenesis and virulence determinants

## Abstract

*Candida albicans* (*C. albicans*) and *Lactiplantibacillus plantarum* subsp. *plantarum* (*L. plantarum*) are frequently identified in various niches, but their dual-species interaction, especially with *C. albicans* in yeast form, remains unclear. This study aimed to investigate the dual-species interaction of *L. plantarum* and *C. albicans*, including proliferation, morphology, and transcriptomes examined by selective agar plate counting, microscopy, and polymicrobial RNA-seq, respectively. Maintaining a stable and unchanged growth rate, *L. plantarum* inhibited *C. albicans* yeast cell proliferation but not hyphal growth. Combining optical microscopy and atomic force microscopy, cell-to-cell direct contact and co-aggregation with *L. plantarum* cells surrounding *C. albicans* yeast cells were observed during dual-species interaction. Reduced *C. albicans* yeast cell proliferation in mixed culture was partially due to *L. plantarum* cell-free culture supernatant but not the acidic environment. Upon polymicrobial transcriptomics analysis, interesting changes were identified in both *L. plantarum* and *C. albicans* gene expression. First, two *L. plantarum* quorum-sensing systems showed contrary changes, with the activation of *lamBDCA* and repression of *luxS*. Second, the upregulation of stress response-related genes and downregulation of cell cycle, cell survival, and cell integrity-related pathways were identified in *C. albicans*, possibly connected to the stress posed by *L. plantarum* and the reduced yeast cell proliferation. Third, a large scale of pathogenesis and virulence factors were downregulated in *C. albicans*, indicating the potential interruption of pathogenic activities by *L. plantarum*. Fourth, partial metabolism and transport pathways were changed in *L. plantarum* and *C. albicans*. The information in this study might aid in understanding the behavior of *L. plantarum* and *C. albicans* in dual-species interaction.

**IMPORTANCE:**

The anti-*Candida albicans* activity of *Lactiplantibacillus plantarum* has been explored in the past decades. However, the importance of *C. albicans* yeast form and the effect of *C. albicans* on *L. plantarum* had also been omitted. In this study, the dual-species interaction of *L. plantarum* and *C. albicans* was investigated with a focus on the transcriptomes. Cell-to-cell direct contact and co-aggregation with *L. plantarum* cells surrounding *C. albicans* yeast cells were observed. Upon polymicrobial transcriptomics analysis, interesting changes were identified, including contrary changes in two *L. plantarum* quorum-sensing systems and reduced cell survival-related pathways and pathogenesis determinants in *C. albicans*.

## INTRODUCTION

Microbial communities widely exist in natural environments (air, soil, and wastewater), various foods (fermented food), and the human body (skin, oral cavity, respiratory tract, gastrointestinal tract, and genital tract) (1–4). Polymicrobial interaction, especially cell-to-cell communication, is essential for microbial cells to coordinate behaviors in various environments and communities (5–7). Fungi and bacteria, which are found living together in a variety of environments and communities, engage in complex interactions that lead to critical microbial behavior ranging from mutualism to antagonism (8). *Candida albicans* and Lactobacilli are frequently identified fungi and bacteria, respectively, in the human oral cavity, gastrointestinal tract, and genital tract (9). Lactobacilli are dominant bacteria in vaginal microbiota and inhabit every major region of the gastrointestinal tract (10, 11). *C. albicans* is an opportunistic pathogen that asymptomatically colonizes the human oral cavity, intestinal tract, genital tract, upper respiratory tract, and skin as a commensal (12). It normally stays as yeast cells in healthy human bodies and only undergoes the yeast-to-hyphae transition in immunocompromised individuals (13, 14). Yeast and hyphae are regularly observed during infection and have distinct functions (15). It has been proposed that both growth forms are important for pathogenicity (16). Hyphae are more invasive than yeast, which is believed to represent the form primarily involved in dissemination (17). In environmental conditions including low pH (<6) and high cell densities (>10^7^ cells/mL), *C. albicans* predominantly grows in the yeast form (15).

The studies on the interaction of Lactobacilli and *C. albicans* had mainly focused on the effect of Lactobacilli, especially their metabolites, including H_2_O_2_, lactic acid, bacteriocin, biosurfactant, exopolysaccharides, and fatty acids, on *C. albicans*, mostly in hyphal form (18, 19). Various studies have shown that the inhibition of *C. albicans* growth by Lactobacilli depends on species and strain type, cell density, interaction time, and environment (19–22). However, some Lactobacillus species and strains had failed to inhibit *C. albicans* growth (23, 24), suggesting the inhibition effect is species- and strain-specific. *C. albicans*, as a polymorphic fungus, is able to rapidly adapt to changing environments by transitioning back and forth from yeast to hypha (25). Besides, with more attention on the hyphal form, researchers have found that the hyphal formation of *C. albicans* could be repressed by the supernatant of certain Lactobacilli (26, 27).

*Lactiplantibacillus plantarum* is present in diverse niches, including the human gastrointestinal tract and genital tract, fermented dairy products, soil, and wastewater (28, 29). In recent years, *L. plantarum* strains from various isolation sites have been adapted to antifungal tests against *C. albicans*, aiming to explore novel antifungal treatment methods. First, some *L. plantarum* strains from different sites of human bodies were examined for their possible use as probiotics to treat *C. albicans* infections. *L. plantarum* MG989 isolated from the vagina of healthy women had the potential to inhibit *C. albicans* growth, suggesting its possible role in helping to clear vulvovaginal candidiasis (VVC) *in vivo* (30). The probiotic supernatant of *L. plantarum* 108 isolated from the human oral cavity, *L. plantarum* CCFM8724 isolated from healthy human feces, *L. plantarum* ATCC 8014, and *L. plantarum* ATCC 14917 significantly inhibited the *Streptococcus mutans* and *C. albicans* mixed-species biofilm formation, providing a new insight into the potential of probiotic *L. plantarum*-based strategies to prevent bacterial-fungal mixed-species biofilms (31–33). Second, specific *L. plantarum* strains from various foods have been tested for possible probiotic and antifungal activity. *L. plantarum* NTU 102 isolated from homemade Korean-style cabbage pickles showed broad spectrum antimicrobial activity, including anti-*C*. *albicans* BCRC 20511 (30). Three *L. plantarum* strains, ATG-K2, ATG-K6, and ATG-K8, isolated from kimchi demonstrated several basic probiotic functions, including a wide range of antibacterial activity, and revealed growth inhibitory effects against *C. albicans* and *Gardnerella vaginalis* (34). In addition, *L. plantarum* had been combined with some chemicals to enhance its anti-*C*. *albicans* activity. Selenium dioxide-treated *L. plantarum* or its cell-free spent broth inhibited the growth of *C. albicans* ATCC 14053 (35).

Collectively, the anti-*C*. *albicans* activity of *L. plantarum* has been explored in the past decades. However, a few aspects had not been considered resulting in the limitation of current studies. First, most studies on the interaction of Lactobacilli and *C. albicans* eliminated the importance of yeast form. The more invasive hyphal form has been commonly recognized as a major contributor to cause pathogenicity. Thus, it is reasonable that hyphal cells have been paid more attention. But yeast form is an inevitable stage *C. albicans* cells would experience, especially in environments in which they co-exist with Lactobacilli (low pH). Second, hyphal growth/proliferation was overlooked in most studies. The influence of Lactobacilli on hyphal formation (yeast-to-hyphae transition) has been emphasized. Third, the intrinsic genetic interaction between *L. plantarum* and *C. albicans* remains unclear. The effect of *C. albicans* on *L. plantarum* had also been omitted. Thus, in this study, we took both *C. albicans* yeast and hyphal form into consideration to explore their interaction with *L. plantarum*. *C. albicans* yeast-*L. plantarum* and *C. albicans* hyphae-*L. plantarum* mixed cultures were set up and their proliferation, morphology, as well as transcriptomics-level changes in both species were investigated.

## RESULTS

### *L. plantarum* inhibits *C. albicans* yeast cells’ proliferation but not hyphal cells

Upon initial assessment by modified agar overlay assay, clear inhibitory zones were observed around either spotted microcolony or streaked culture line of *L. plantarum* (Fig. S1), suggesting the inhibition of *L. plantarum* on *C. albicans* proliferation. Thus, with single culture as a control, the proliferation of *C. albicans* and *L. plantarum* in mixed culture was examined by colony-forming unit (CFU) counting on respective selective agar plates. Considering filamentation is a distinct trait of *C. albicans*, yeast and hyphal cells were examined separately in the interaction with *L. plantarum*.

**Fig 1:**
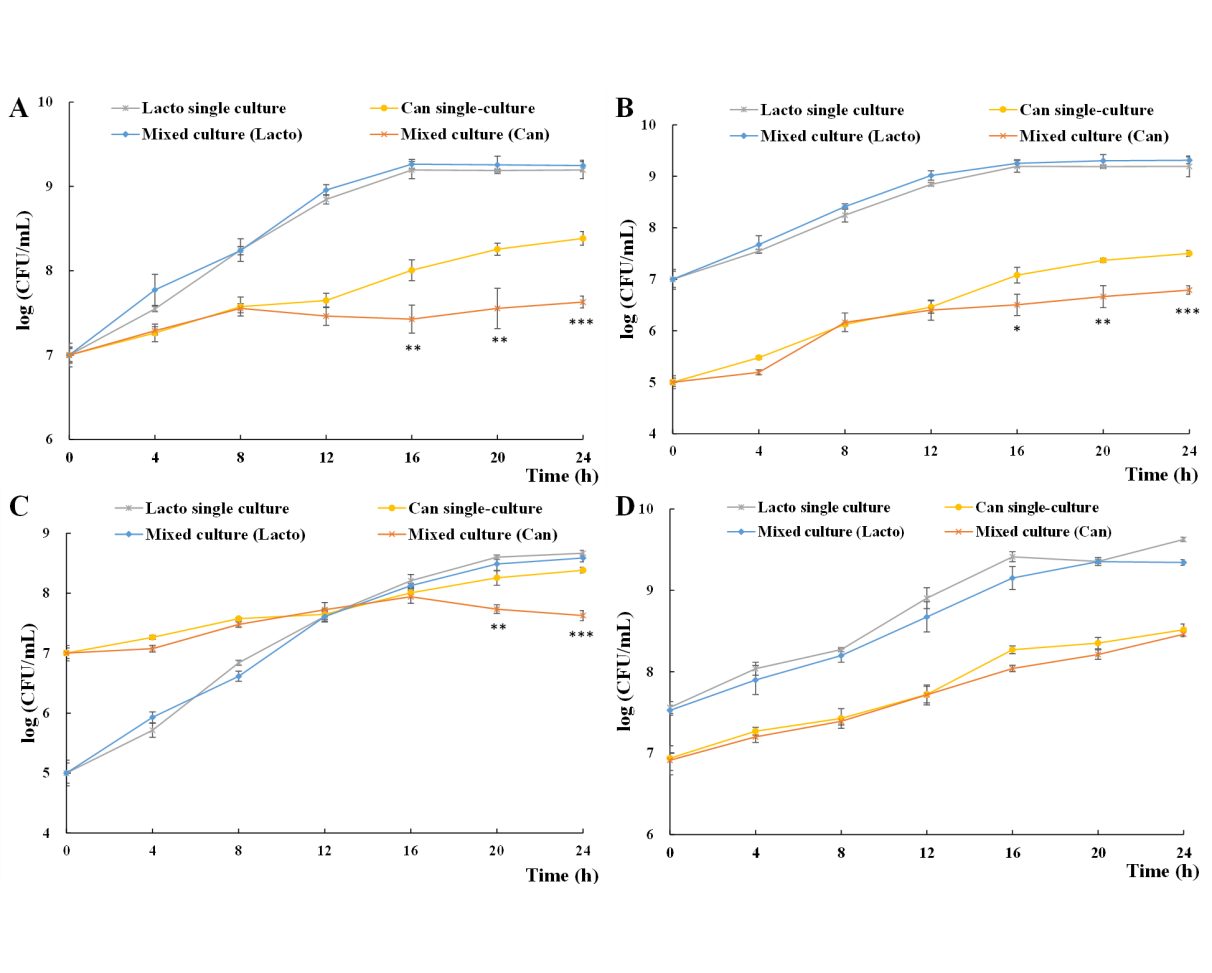
*L. plantarum* inhibits *C. albicans* yeast cell proliferation at an *L. plantarum-C. albicans* ratio of 1:1 (**A**), 100:1 (**B**), and 1:100 (**C**) and failed to induce reduced hyphal growth (**D**). All experiments were conducted in biological triplicates. *P < 0.05; **P < 0.01; and ***P < 0.001.

In the mixed culture of *L. plantarum* and *C. albicans* yeast cells (Fig. 1A through C), the growth of *L. plantarum* was stable and consistent with that in the corresponding single culture, indicating *L. plantarum* cell proliferation was not influenced by *C. albicans* yeast cells. The growth of *C. albicans* yeast cells in mixed culture remained stable and uninfluenced within 12 h in all groups. However, in mixed culture, yeast cell proliferation was suspended when *L. plantarum* entered the stationary phase (16 h in 1:1 and 100:1 groups and 20 h in 1:100 group), resulting in a decreased yeast cell number (80.45%–82.49% less at 24 h) compared with single culture. The significant inhibition appeared at 16 h in the 1:1 and 1:100 groups (Fig. 1A and B) and 20 h in the 100:1 group (Fig. 1C), corresponding with *L. plantarum* stationary phases. Concerning inhibition rate, in the 1:1 group, 73.66% (7.46 × 10^7^ CFU/mL), 80.14% (1.44 × 10^8^ CFU/mL), and 82.34% (1.99 × 10^8^ CFU/mL) reduction were recorded at 16, 20, and 24 h in *C. albicans* yeast cell proliferation, respectively. In the 100:1 group, 73.37% (8.80 × 10^6^ CFU/mL), 80.31% (1.88 × 10^7^ CFU/mL), and 80.45% (2.55 × 10^7^ CFU/mL) reduction were recorded at 16, 20, and 24 h in *C. albicans* yeast cell proliferation, respectively. In the 1:100 group, 69.89% (1.26 × 10^8^ CFU/mL) and 82.49% (1.99 × 10^8^ CFU/mL) reduction were recorded at 20 and 24 h in *C. albicans* yeast cell proliferation, respectively. The results indicated that the inhibition of *L. plantarum* on *C. albicans* yeast cell proliferation is dependent on the cell density of *L. plantarum*.

Hyphal cells are more robust and adapt to various environments (36). In the mixed culture of *L. plantarum* and *C. albicans* hyphal cells, the proliferation of both strains was not influenced (Fig. 1D). Considering *C. albicans* stays as yeast cells in the oral cavity, intestinal tract, and genital tract of healthy human bodies, where they co-exist with *L. plantarum*, it is important to explore their interaction and how they stay (13, 14). Thus, the interaction between *L. plantarum* and *C. albicans* yeast cells was further explored.

### Co-aggregation occurs during dual-species interaction

**Fig 2:**
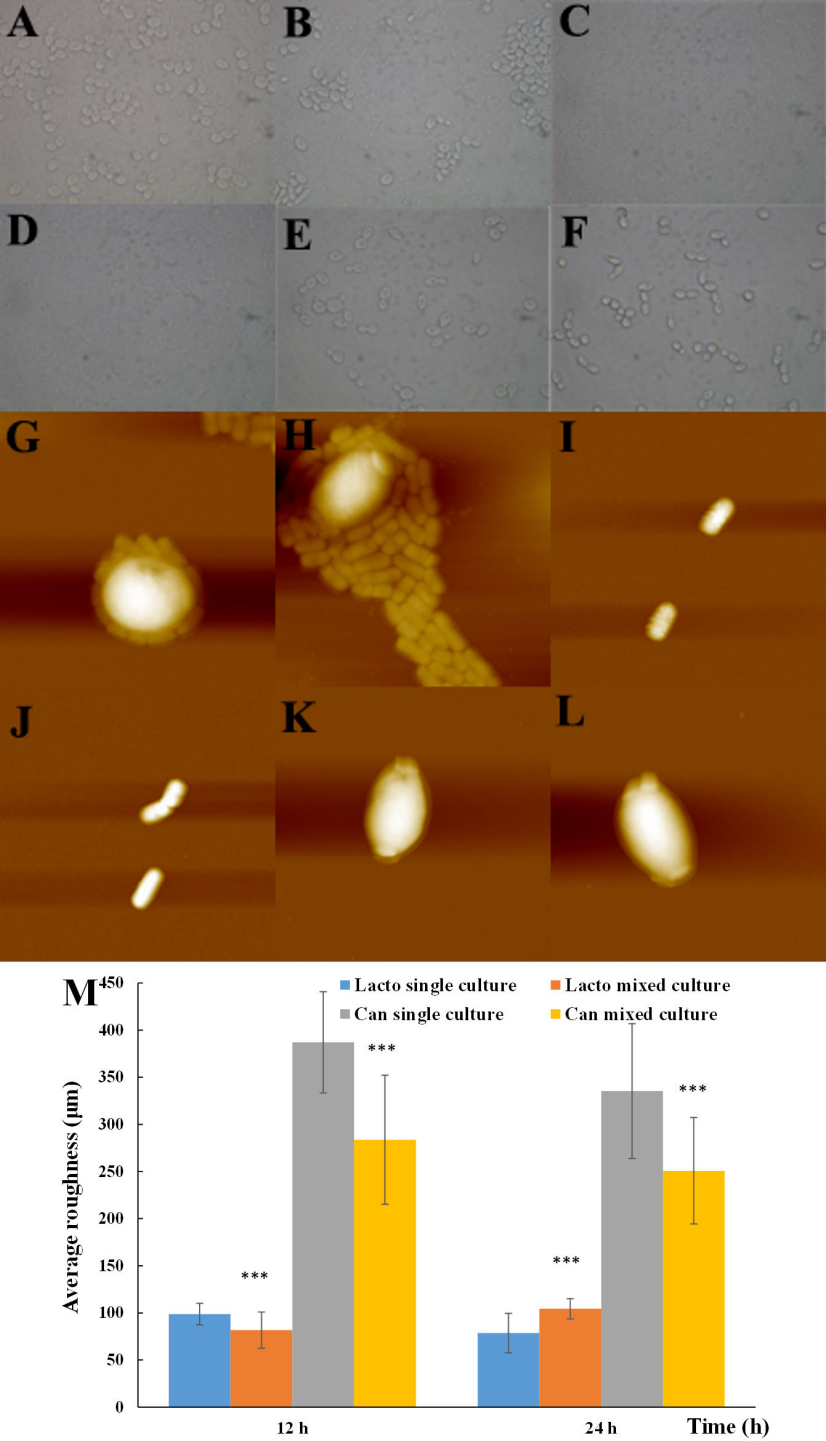
The morphology of *L. plantarum* and *C. albicans* yeast cells under optical microscope (**A–E**) and atomic force microscope (**F–K**). as well as surface roughness (**M**). (A and G) LhCa_12 h, (B and H) LhCa_24 h, (C and I) Lh_12 h, (D and J) Lh_24 h, (E and K) Ca_12 h, and (F and L) Ca_24 h. All experiments were conducted in biological triplicates. *P < 0.05; **P < 0.01; and ***P < 0.001.

The morphology of *L. plantarum* and *C. albicans* yeast cells was observed under an optical microscope and atomic force microscope (AFM) (Fig. 2). No significant difference in cell shape was identified in optical microscopic images of single and mixed cultures (Fig. 2A through F). Interestingly, in the 24-h mixed culture, more *C. albicans* yeast cell aggregates surrounded by a few *L. plantarum* cells were observed (Fig. 2B). Such a phenomenon was also observed in the AFM images of mixed cultures, with single cultures as controls (Fig. 2G through L), demonstrating direct cell-cell contact and interaction between *L. plantarum* and *C. albicans* yeast cells. Each *C. albicans* yeast cell was surrounded by multiple *L. plantarum* cells (Fig. 2G and H). The surface roughness of *L. plantarum* and *C. albicans* yeast cells was calculated based on the 3D images from AFM. The surface roughness of yeast cells was three times higher than that of *L. plantarum* cells and showed a significant decrease in mixed culture (Fig. 2I).

### Role of cell-free culture supernatant in reduced *C. albicans* yeast cell proliferation

**Fig 3:**
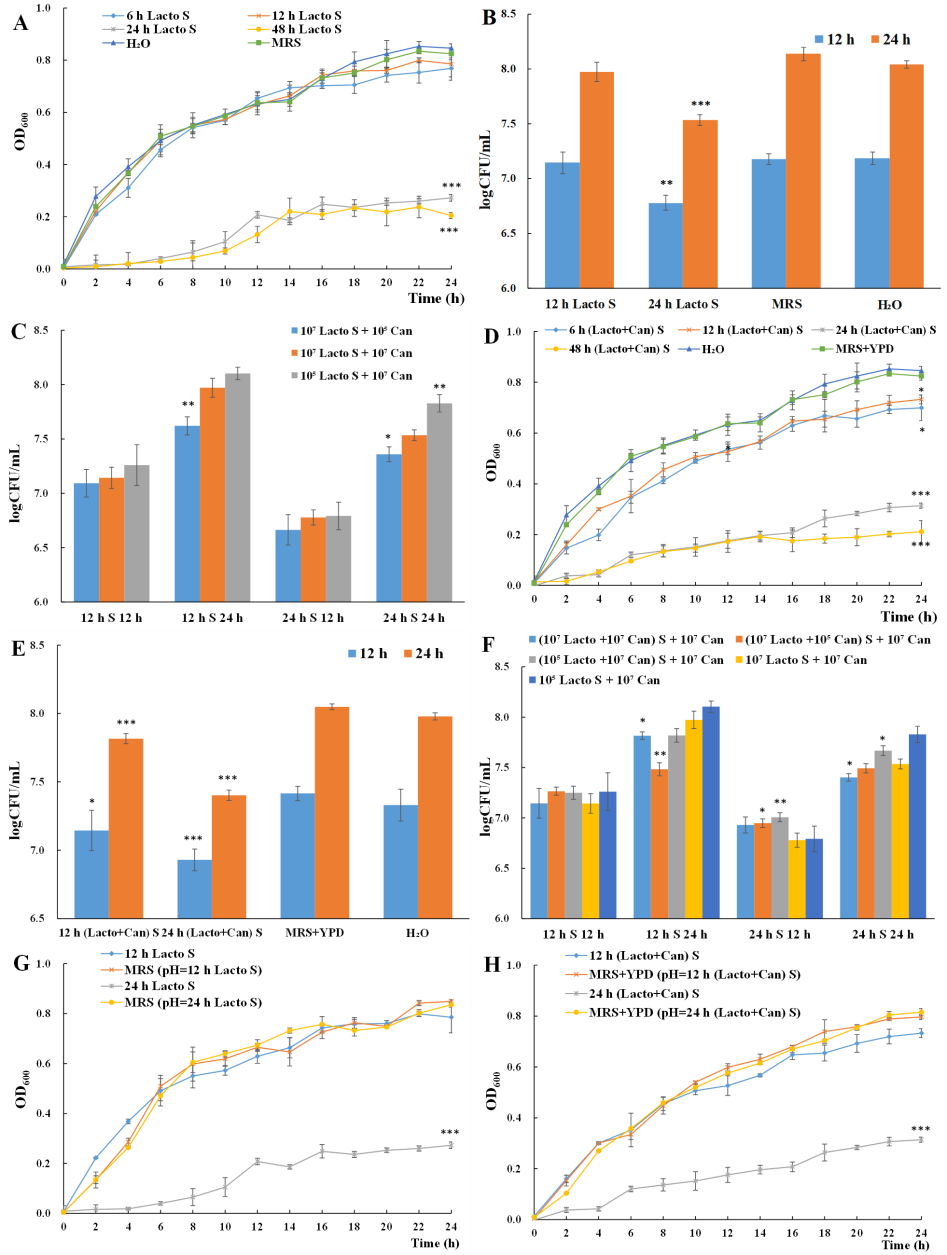
Role of CFCS in reduced *C. albicans* yeast cell proliferation. (A–C) Yeast cell proliferation in groups supplemented with CFCS from *L. plantarum* single culture examined by OD_600_ (**A**) and CFU (**B**), with different initial concentrations (**C**). (D–F) Yeast cell proliferation in groups supplemented with CFCS from mixed culture examined by OD_600_ (**D**) and CFU (**E**), with different initial concentrations (**F**). (G and H) *C. albicans* yeast cell proliferation in CFCS from *L. plantarum* single culture (**G**) and mixed culture (**H**) mimicking acidified media. All experiments were conducted in biological triplicates. *P < 0.05; **P < 0.01; and ***P < 0.001.

With De Man Rogosa Sharpe (MRS) broth and distilled H_2_O as control groups, cell-free culture supernatant (CFCS) collected from 6-, 12-, 24-, and 48-h *L*. *plantarum* culture (6 h Lacto S, 12 h Lacto S, 24 h Lacto S, and 48 h Lacto S) was added in *C. albicans* yeast culture to examine the change in yeast cell proliferation. Different initial concentrations (10^7^ and 10^5^ CFU/mL) and ratios (1:1, 1:100, and 100:1) of *L. plantarum* and *C. albicans* were included in this experiment. Here, the growth of *C. albicans* yeast cells with the supplement of CFCS collected from *L. plantarum* culture with an initial concentration of 10^7^ CFU/mL is shown as a representative (Fig. 3). The yeast cell proliferation in groups supplemented with 6 h Lacto S and 12 h Lacto S (Fig. 3A) remained unchanged compared to control groups. Significant repression of yeast cell proliferation was observed in groups supplemented with 24 h Lacto S and 48 h Lacto S (Fig. 3A). The culturable yeast cell numbers at 12 and 24 h in groups supplemented with 12 h Lacto S (no inhibition) and 24 h Lacto S (inhibition) were further examined (Fig. 3B). Consistently, no change was observed in the group supplemented with 12 h Lacto S, while significant reduction (59.84% at 12 h and 74.92% at 24 h) was recorded in the group supplemented with 24 h Lacto S. Thus, CFCS from *L. plantarum* culture played a role in the reduced *C. albicans* yeast cell proliferation but was maturity dependent. In addition, the initial concentration of *C. albicans* was positively related to the inhibition rate (Fig. 3C; Fig. S2).

For the groups supplemented with CFCS collected from 6-, 12-, 24-, and 48-h mixed culture [6 h (Lacto + Can) S, 12 h (Lacto + Can) S, 24 h (Lacto + Can) S, and 48 h (Lacto + Can) S], MRS-yeast peptone dextrose (YPD) broth and distilled H_2_O served as control groups. Similarly, significant repression of yeast cell proliferation was observed in groups supplemented with 24 h (Lacto + Can) S and 48 h (Lacto + Can) S (Fig. 3D). Surprisingly, 6 h (Lacto + Can) S and 12 h (Lacto + Can) S also showed significant inhibition on yeast cell proliferation although to a much lower level than 24 h (Lacto + Can) S and 48 h (Lacto + Can) S. The culturable yeast cell numbers at 12 and 24 h in groups supplemented with 12 h (Lacto + Can) S and 24 h (Lacto + Can) S were further examined (Fig. 3E). The inhibition rate was 67.32% at 12 h and 67.32% at 24 h for 24 h (Lacto + Can) S group and 46.39% at 12 h and 46.39% at 24 h for 12 h (Lacto + Can) S group. In the groups with different initial concentrations (10^7^ and 10^5^ CFU/mL) and ratios (1:1, 1:100, and 100:1) of *L. plantarum* and *C. albicans*, a similar observation was found (Fig. S3). In comparison with CFCS collected from *L. plantarum* single culture, corresponding CFCS collected from mixed culture showed stronger inhibition on yeast cell proliferation (Fig. 3F), indicating specific metabolite accumulation during the interaction between *L. plantarum* and *C. albicans* yeast cells.

### CFCS mimicking acidified medium is not sufficient to cause reduced *C. albicans* yeast cell proliferation

Acid production is a typical trait of *L. plantarum,* which may elicit an adverse environment for *C. albicans* growth (37, 38). In order to test the role of acidic environment in reduced *C. albicans* yeast cell proliferation in mixed culture, the pH value of CFCS was determined. The pH values of MRS broth, MRS-YPD, 12 h Lacto S, 24 h Lacto S, 12 h (Lacto + Can) S, and 24 h (Lacto + Can) S were 5.64, 4.17, 3.88, 5.80, 4.44, and 3.82, respectively. The pH of CFCS collected from *L. plantarum* single culture showed no significant difference from that collected from the mixed culture. However, CFCS from mixed culture showed stronger inhibition on *C. albicans* yeast cell proliferation. Thus, pH might not play a key role. For verification, CFCS mimicking acidified medium was prepared by adjusting the pH of MRS and MRS-YPD broth to the same as CFCS and was used as a growth medium for *C. albicans* yeast cells. Undoubtedly, CFCS mimicking acidified medium was not sufficient to cause reduced *C. albicans* yeast cell proliferation (Fig. 3G and H). *C. albicans* yeast cells were able to adapt to acidic environment yield by *L. plantarum*.

### Transcriptomes of *L. plantarum* and *C. albicans* yeast

**Fig 4:**
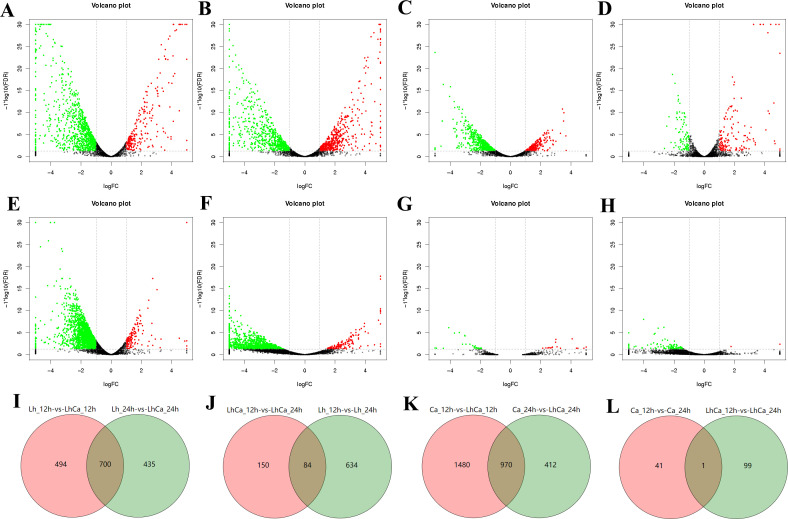
Volcano plots of *L. plantarum* comparative group 1: Lh_12 h vs LhCa_12 h (**A**), group 2: Lh_24 h vs LhCa_24 h (**B**), group 3: Lh_12 h vs Lh_24 h (**C**), group 4: LhCa_12 h vs LhCa_24 h (**D**), and *C. albicans* comparative group 1: Ca_12 h vs LhCa_12 h (**E**), group 2: Ca_24 h vs LhCa_24 h (**F**), group 3: Ca_12 h vs Ca_24 h (**G**), group 4: LhCa_12 h vs LhCa_24 h (**H**), as well as Venn map of overlapped and unique DEGs between comparative groups: *L. plantarum* group 1 vs 2 (**I**), 3 vs 4 (**J**) and *C. albicans* group 1 vs 2 (**K**), 3 vs 4 (L).

To explore the intrinsic change in *L. plantarum* and *C. albicans* yeast cells, single and mixed cultures were collected at 12 and 24 h in biological triplicates and designated as Lh_12 h (12-h *L*. *plantarum* single culture), Lh_24 h (24-h *L*. *plantarum* single culture), Ca_12 h (12-h *C*. *albicans* single culture), Ca_24 h (24-h *C*. *albicans* single culture), LhCa_12 h (12-h mixed culture), and LhCa_24 h (24-h mixed culture). To represent the status before and after reduced *C. albicans* yeast cell proliferation, 12- and 24-h samples were selected. The samples were adapted to RNA-sequencing (RNA-seq) and downstream bioinformatics analysis. The reads from Lh_12 h and Lh_24 h were mapped to the reference genome of *L. plantarum* WCFS1, and the reads from Ca_12 h and Ca_24 h were mapped to the reference genome of *C. albicans* SC5314. Especially, reads from LhCa_12 h and LhCa_24 h were mapped to both reference genomes and annotated separately. Fragments per kilobase of transcript per million mapped reads (FPKM) value was used to determine the relative gene expression level of each sample. To identify differentially expressed genes (DEGs), eight comparative groups were investigated, including four groups comparing *L. plantarum* genes (group 1: Lh_12 h vs LhCa_12 h, group 2: Lh_24 h vs LhCa_24 h, group 3: Lh_12 h vs Lh_24 h, and group 4: LhCa_12 h vs LhCa_24 h) and four groups comparing *C. albicans* genes (group 1: Ca_12 h vs LhCa_12 h, group 2: Ca_24 h vs LhCa_24 h, group 3: Ca_12 h vs Ca_24 h, and group 4: LhCa_12 h vs LhCa_24 h). Relatively large amounts of DEGs were identified in each group (Fig. 4A through H; Tables S1 and S2). Considering the growth deficiency of *C. albicans* yeast in mixed culture appeared at 24 h but not at 12 h, overlapped and unique DEGs between comparative groups (*L. plantarum* group 1 vs 2, 3 vs 4 and *C. albicans* group 1 vs 2, 3 vs 4) were determined to specifically focus on genes potentially inducing such difference (Fig. 4I through L; Table S3). A total of 435 and 412 unique DEGs were identified in *L. plantarum* group 2 (Fig. 4I) and *C. albicans* group 2 (Fig. 4K), respectively, representing the key genes changed in 24-h mixed culture. Regarding potential genes causing reduced *C. albicans* yeast cell proliferation, 150 and 99 DEGs were uniquely changed in *L. plantarum* group 4 (Fig. 4J) and *C. albicans* group 4 (Fig. 4L), respectively. The DEGs in each group were subsequently adapted to annotation against the non-redundant (Nr) database, the Gene Ontology (GO) database, and the Kyoto Encyclopedia of Genes and Genomes (KEGG) pathway database, as well as enrichment analysis.

### Key DEGs alteration in *L. plantarum* in 12-h interactome

A total of 1,194 genes (234 upregulated and 960 downregulated genes) in *L. plantarum* showed significant expression change in group 1, representing 12-h interactome with *C. albicans* (Fig. S1). Based on their fold change and encoding protein, the unique DEGs in *L. plantarum* group 1 were compared to group 2 and analyzed (Table S3). Quorum sensing (QS) has been documented as a cell-cell communication system that coordinates interactions both within one species and between different species (39). Signal transduction is also a key trait in cell-cell communication. Thus, we paid more attention to the genes involved in quorum-sensing system and signal transduction. In addition, genes that showed no expression in one sample but highly expressed in the other sample within the same group were more likely to contribute to the microbial interaction. For unique DEGs in group 1, 69 genes showed upregulation, including three signaling factors (*citF*: 5.47-fold, *citE*: 4.92-fold, and *mae*: 2.40-fold), and 449 genes showed downregulation, including 23 genes that stopped expression in sample LhCa_12 h.

**Fig 5:**
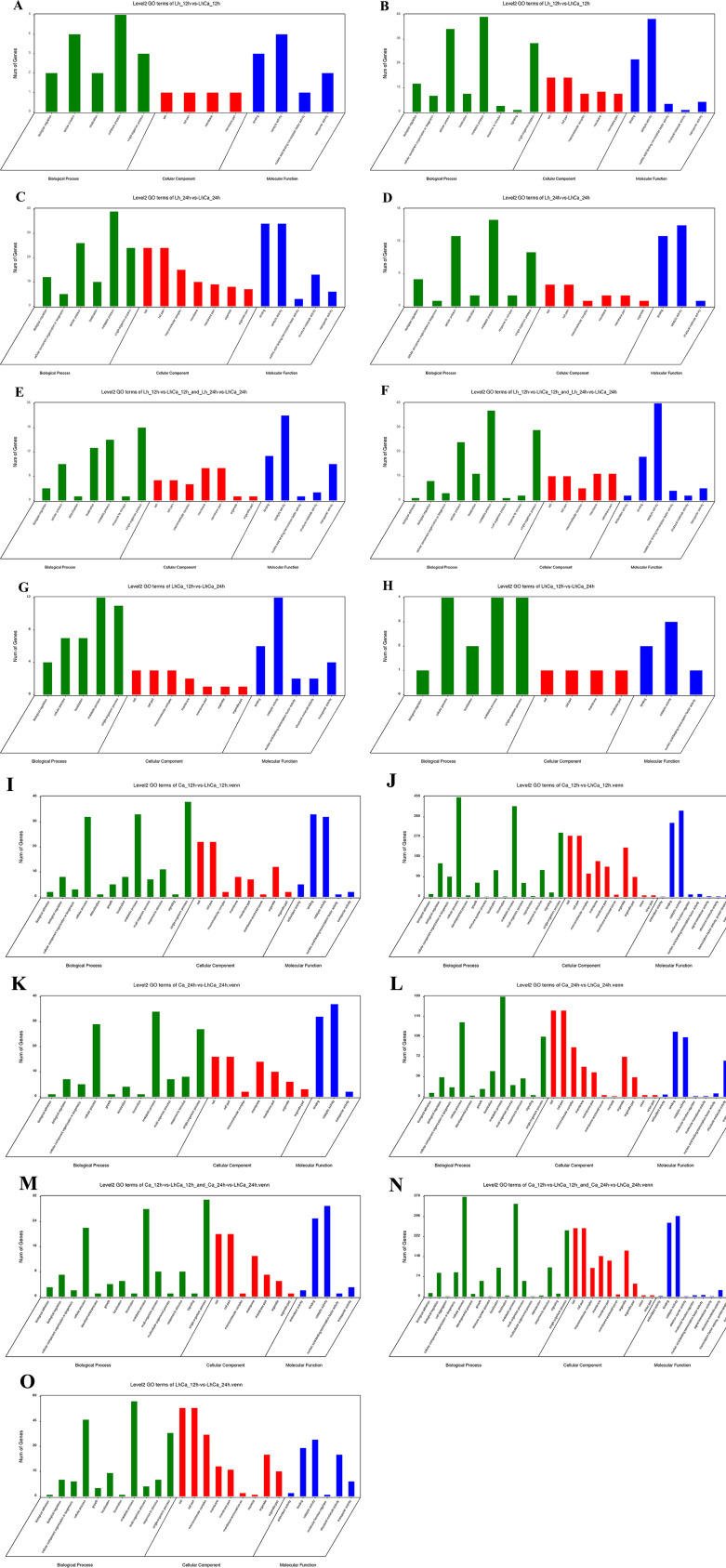
Significantly enriched GO terms for unique DEGs in *L. plantarum* groups 1 (**A and B**), 2 (**C and D**), and 4 (**E and F**), respectively, and overlapped DEGs in *L. plantarum* group 1 vs 2 (**G and H**), as well as in *C. albicans* groups 1 (**I and J**), 2 (**K and L**), and 4 (**M and N**), respectively, and overlapped DEGs in *C. albicans* group 1 vs 2 (**O**).

Next, the unique DEGs were adapted to GO term enrichment analysis. Up and downregulated DEGs were analyzed separately. Uniquely upregulated DEGs in group 1 (Fig. 5A) were significantly enriched in “carbohydrate binding,” “carbohydrate transmembrane transporter activity,” and “carbohydrate transmembrane transporter activity” in molecular function (MF) and “D-ribose metabolic process” and “pentose metabolic process” in biological process (BP). Uniquely downregulated DEGs in group 1 (Fig. 5B) were significantly enriched in “DNA polymerase complex” and “transferase complex” in cellular component (CC), “ligase activity, forming carbon-oxygen bonds,” “ligase activity, forming aminoacyl-tRNA and related compounds,” and “nucleotidyltransferase activity” in MF, “cellular component organization or biogenesis,” “single-organism cellular process,” and “tRNA metabolic process” in BP.

**Fig 6:**
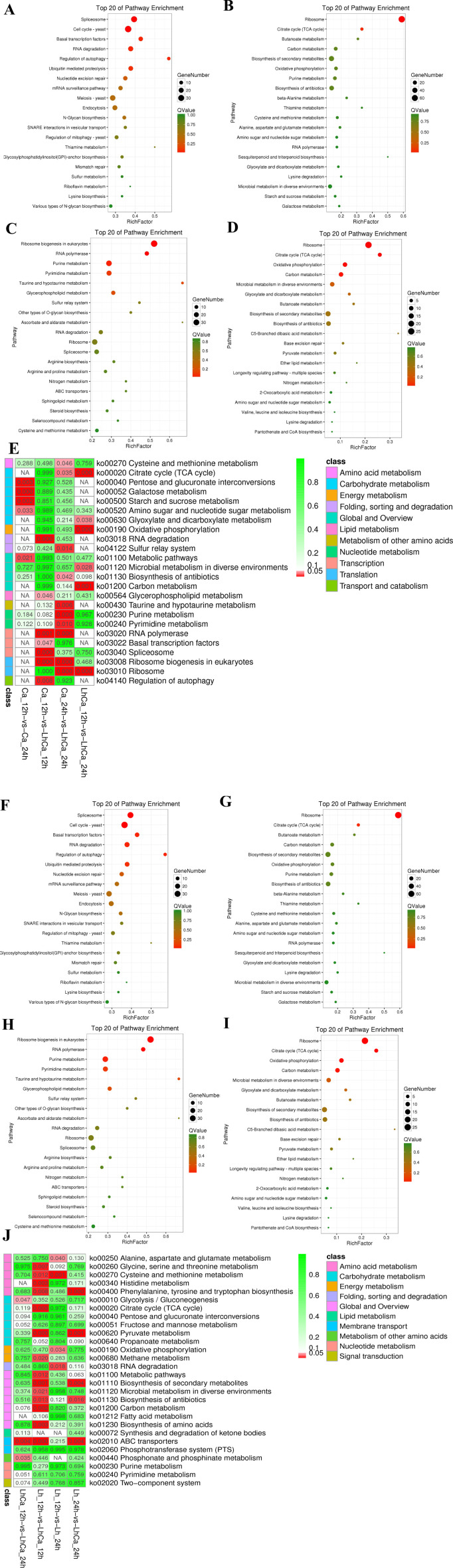
Significantly enriched KEGG pathways for unique DEGs in *L. plantarum* groups 1 (**A**), 2 (**B**), and 4 (**C**), respectively, and overlapped DEGs in *L. plantarum* group 1 vs 2 (**D**), and comparison among groups (**E**), as well as in *C. albicans* groups 1 (**F**), 2 (**G**), and 4 (**H**), respectively, and overlapped DEGs in *C. albicans* group 1 vs 2 (**I**), and comparison among groups (**J**).

Additionally, the unique DEGs were adapted to KEGG pathway enrichment analysis. A total of 10 pathways were significantly enriched in unique DEGs in group 1 (Fig. 6A), including “biotin metabolism,” “aminoacyl-tRNA biosynthesis,” “carbon metabolism,” “microbial metabolism in different environments,” “glycine, serine, and threonine metabolism,” “methane metabolism,” “fatty acid metabolism,” “RNA polymerase,” “selenocompound metabolism,” and “fatty acid biosynthesis.”

### Key DEGs alteration in *L. plantarum* in 24-h interactome

**Fig 7:**
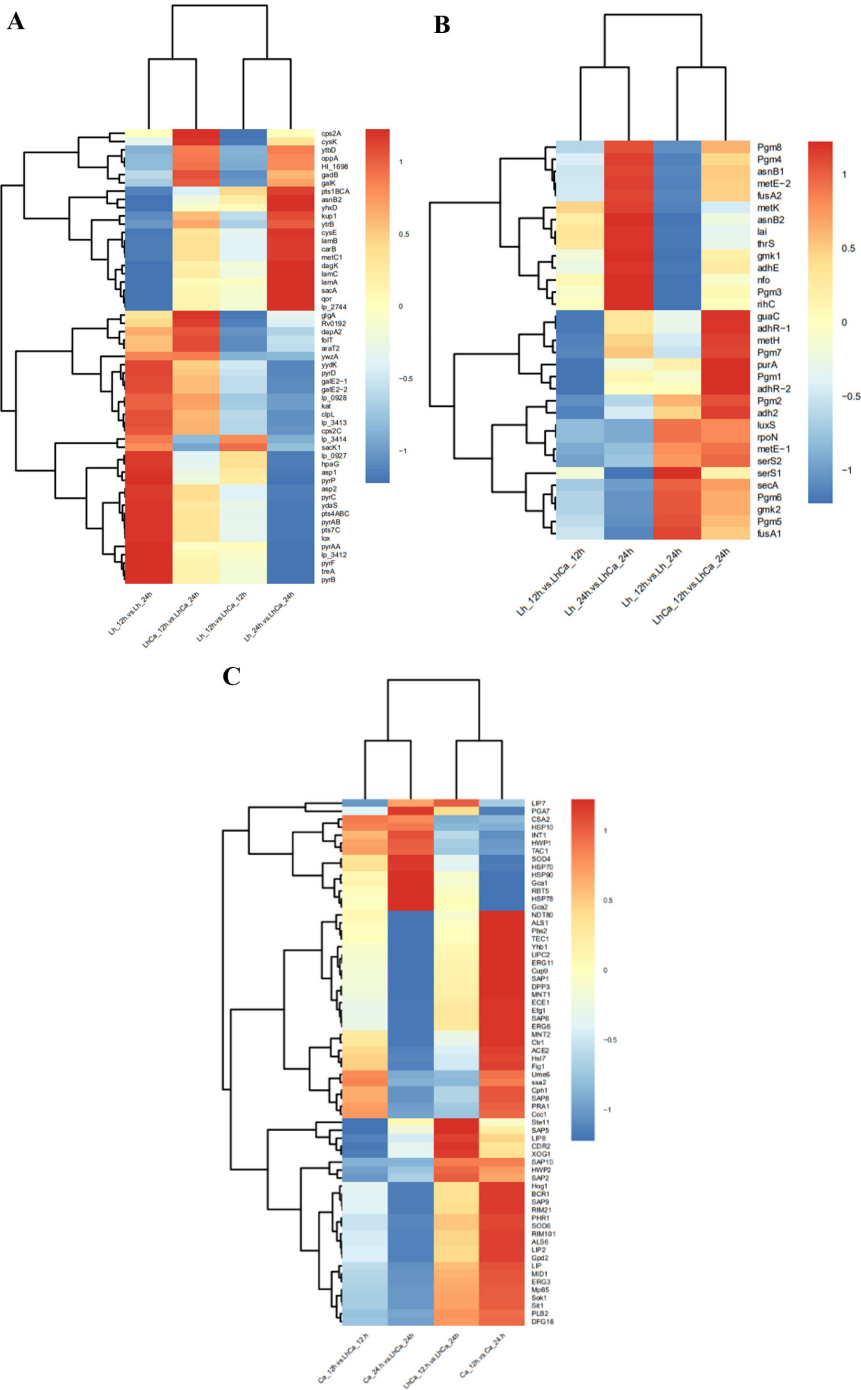
Expression level changes of the Lam system (**A**) and luxS/AI-2 (**B**) regulated genes in *L. plantarum* and pathogenicity-related genes (**C**) in *C. albicans* among the four comparative groups.

A total of 1,135 genes (477 upregulated and 658 downregulated genes) in *L. plantarum* showed significant expression change in group 2, representing 24-h interactome with *C. albicans* (Fig. S1). Based on their fold change and encoding protein, the unique DEGs in *L. plantarum* group 2 were compared to group 1 and analyzed (Table S3). Among the unique DEGs in group 2, 312 genes were upregulated, including nine signaling factors (*lamB*: 21.71-fold, *lamC*: 6.87-fold, *lamA*: 16.56-fold, *sip1*: 3.56-fold, *hpk3*: 2.64-fold, *sip3*: 2.23-fold, *zmp1*: 3.73-fold, *ica1*: 3.27-fold, *lytN*: 20.25-fold, and *isaA*: 564.18-fold), and 147 genes were downregulated. Genes (*lamA*, *lamB*, *lamC*, and *lamD*) involved in the two-component regulatory quorum-sensing system Lam were uniquely upregulated in group 2, indicating *C. albicans* yeast cells induced the activation of *L. plantarum* QS system Lam in mixed culture at 24 h but not at 12 h. The expression of downstream genes regulated by the Lam system also changed correspondingly, including membrane protein-encoding genes (*ydaS*: 0.06-fold, *folT*: 4.79-fold, and *ywzA*: 0.05-fold) and stress response genes (*asp1*: 0.18-fold, *asp2*: 0.01-fold, and *kat*: 0.43-fold) (Fig. 7A). In the previous report, deletion of *lamA* gene showed no difference in growth rate and cell or colony morphology but acquired reduced adherence to glass surfaces (40). Thus, the *lamA* gene was closely related to cell adherence ability, and its upregulation might contribute to the adherence of *L. plantarum* cells to *C. albicans* yeast cells in mixed culture observed under microscopy.

For unique DEGs in group 2, upregulated genes (Fig. 5C) were significantly enriched in “ribosome,” “organelle,” “cytoplasm,” “ribonucleoprotein complex,” and “macromolecular complex” in CC, “RNA binding,” “structural molecule activity,”and “nucleic acid binding” in MF, “gene expression,” “posttranscriptional regulation of gene expression,” and “carbohydrate derivative transport” in BP. While downregulated genes (Fig. 5D) were significantly enriched in “transferase activity, transferring pentosyl groups,” “transferase activity, transferring pentosyl groups,” and “heterocyclic compound binding” in MF, and “nucleobase-containing compound metabolic process,” “UMP biosynthetic process,” and “pyrimidine nucleoside monophosphate metabolic process” in BP. Only three pathways were significantly enriched in unique DEGs in group 2 (Fig. 6B), including “ribosomes,” “terpenoid backbone biosynthesis,” and “aminoacyl-tRNA biosynthesis.”

### Key DEGs’ alteration in *L. plantarum* in 12- and 24-h interactome

Regarding overlapped genes in group 1 and 2, representing 12- and 24-h interactome with *C. albicans*, 165 genes were upregulated, including eight signaling factors (*pstF*: 102.54-fold, *pstE*: 36.15-fold, *mleS*: 16.31-fold, *ifcA*: 15.60-fold, *isaA*: 564.18-fold, and *iap*: 306.94-fold), and 511 genes were downregulated including *luxS* gene (0.07-fold). The *LuxS* gene is the key component of the *LuxS*/autoinducer-2 (AI-2) QS system. The downregulation of the *luxS* gene in mixed culture at both 12 and 24 h indicated the repression of the *L. plantarum luxS*/AI-2 QS system by *C. albicans* yeast. Genes involved in the AI-2 production pathway were also downregulated in mixed culture (Fig. 7B).

Concerning GO terms, upregulated DEGs (Fig. 5E) were significantly enriched in “transmembrane transporter activity,” “transporter activity,” and “oxidoreductase activity, acting on the aldehyde or oxo group of donors” in MF, and “carbohydrate transport,” “organic substance transport,” and “localization” in BP. Downregulated DEGs (Fig. 5F) were significantly enriched in “lyase activity,” “antioxidant activity,” and “ion transmembrane transporter activity” in MF and “single-species metabolic process,” “cation transport” and “amine transport” in BP.

In overlapped DEGs in group 1 vs 2, 10 pathways were significantly enriched (Fig. 6C), including “ABC transporter,” “tyrosine and tryptophan biosynthesis,” “pyruvate metabolism,” “secondary metabolite biosynthesis,” “antibiotic biosynthesis,” “TCA cycle,” “histidine metabolism,” “sphingolipid metabolism,” “amino acid biosynthesis,” and “propionic acid metabolism.”

### Key DEGs during maturation of *L. plantarum* from 12 to 24 h

A total of 718 genes (181 upregulated and 537 downregulated genes) in *L. plantarum* showed significant expression change in group 3, representing its maturation from 12 to 24 h (Fig. S1). While 234 genes (145 upregulated and 89 downregulated genes) in *L. plantarum* showed significant expression change in group 4, representing its interactome from 12 to 24 h with *C. albicans*. Based on their fold change and encoding protein, the unique DEGs in *L. plantarum* group 4 were compared to group 3 and analyzed (Table S3). In group 4, 122 and 63 unique DEGs were up and downregulated, respectively. Among them, eight signaling factors (*lamB*: 4.44-fold, *lamC*: 2.40-fold, *pstF*: 3.61-fold, *pstE*: 4.01-fold, *lamA*: 2.59-fold, *isaA*: 30.75-fold, *iap*: 9.57-fold, and *lytN*: 6.23-fold) were upregulated.

Regarding GO terms, upregulated DEGs (Fig. 5G) were significantly enriched in “carbohydrate transmembrane transporter activity,” “hydrolase activity,” “amylase activity,” and “intramolecular transferase activity” in MF. The downregulated DEGs (Fig. 5H) were significantly enriched in “ligase activity” in MF, and “nucleotide metabolism and biosynthesis process,” “phosphoribose metabolism process,” and “carbohydrate derivative metabolism and biosynthesis process” in BP.

A total of five pathways were significantly enriched in unique DEGs in group 4 (Fig. 6D), including “Pyrimidine metabolism,” “ABC transporter,” “phosphate and phosphite metabolism,” “Glycolysis/Gluconeogenesis,” and “pentose and glucuronate interconversions.” In addition, the enriched KEGG pathways were compared among the four groups (Fig. 6E).

### Key DEGs’ alteration in *C. albicans* in 12-h interactome

A total of 2,450 genes (140 upregulated and 2,310 downregulated genes) in *C. albicans* showed significant expression change in group 1, representing 12-h interactome with *L. plantarum* (Fig. S1). Based on their fold change and encoding protein, the unique DEGs in *C. albicans* group 1 were compared to group 2 and analyzed (Table S3). Considering *C. albicans* is an opportunistic fungal pathogen of humans (14), the elements contributing to its pathogenesis were paid more attention as key DEGs might be changed by the existence of *L. plantarum* cells. Representing the key genes during the interaction of *C. albicans* and *L. plantarum* within 12 h, 80 upregulated and 1,412 downregulated DEGs were uniquely identified in *C. albicans* group 1. Regarding upregulated genes, 11 stress response factors (*nag3*: 8.16-fold, *ssa2*: 6.51-fold, *trr1*: 5.65-fold, *crz1*: 0.71-fold, *cat1*: 2.54-fold, *tsa1*/*1b*: 2.23-fold, *glr1*: 2.23-fold, *iqg1*: 2.09-fold, *rpo21*: 2.06-fold, and *spbc1683.03c*: 2.04-fold) were identified, and *dan4* gene was not expressed in sample Ca_12 h. Among downregulated genes, a total of 19 genes were not expressed in LhCa_12 h, indicating they were totally repressed in mixed culture. In addition, virulence determinants *dfg16* (0.32-fold) and *rim 21* (0.44-fold) responsible for pH sensing, *cdr2* (0.42-fold) and *upc2* (0.89-fold) contributing to drug resistance, *xog1* (0.46-fold) encoding exoglucanase involved in immune evasion, *sap2* (0.16-fold), *sap5* (0.38-fold), and *sap10* (0.27-fold) encoding secreted aspartyl proteinases were uniquely downregulated DEGs in *C. albicans* group 1. It suggested the existence of *L. plantarum* cells influenced the pH sensing, drug resistance, and immune evasion of *C. albicans* at 12 h.

The unique DEGs were adapted to GO term enrichment analysis. Up and downregulated DEGs were analyzed separately. Uniquely upregulated DEGs in group 1 (Fig. 5I) were significantly enriched in “cell wall,” “microbodies,” “external encapsulation structures,” “cell periphery,” and “cytoplasm” in CC, “nucleotide binding,” “oxidoreductase activity,” and “antioxidant activity” in MF, and “monotypic metabolic processes,” “hydrogen peroxide metabolism processes,” and “NADP metabolic processes” in BP. Uniquely downregulated DEGs in group 1 (Fig. 5J) were significantly enriched in “membrane-wrapped organelle” in CC and “intracellular macromolecular metabolism process” and “nucleic acid metabolism process” in BP.

The unique DEGs were also adapted to KEGG pathway enrichment analysis (Fig. 6). Significantly enriched pathways in unique DEGs in group 1 (Fig. 6F) included “spliceosome,” “cell cycle,” “basal transcription factors,” “RNA degradation,” “autophagy regulation,” “ubiquitin-mediated proteolysis,” “nucleotide excision repair,” “mRNA monitoring pathway,” “meiosis,” “cell cycle,” and “endocytosis.”

### Key DEGs’ alteration in *C. albicans* in 24-h interactome

A total of 1,382 genes (130 upregulated and 1,252 downregulated genes) in *C. albicans* showed significant expression change in group 2, representing 24-h interactome with *L. plantarum* (Fig. S1). Based on their fold change and encoding protein, the unique DEGs in *C. albicans* group 2 were compared to group 1 and analyzed (Table S3). A total of 70 upregulated and 348 downregulated DEGs, representing the key genes in the interaction from 12 to 24 h, were uniquely identified in *C. albicans* group 2. Among upregulated genes, eight stress response-related genes (*adh2*: 8.03-fold, *dac1*: 6.86-fold, *thi4*: 6.61-fold, *cdc37*: 6.23-fold, *cmk1*: 5.08-fold, *hsp70*: 3.47-fold, *hsp78*: 4.63-fold, *ammecr1*: 3.41-fold, and *pgk1*: 3.26-fold) and nutrient acquisition-related genes (*rbt5*: 3.21-fold and *pga7*: 3.46-fold) were included and *nsa2* gene (0.02-fold) showed no expression in sample Ca_24 h. Showing no expression in sample LhCa_24 h, 41 genes, including *pra1* encoding zinc acquisition, were possibly repressed by the stress posed by *L. plantarum* cells. Concerning downregulated DEGs, virulence determinants including phenotypic switch-related genes *cph1* (0.10-fold, transcription factor) and *tec1* (0.15-fold, filamentation inducer, biofilm formation) and transcription factor *ace2* (0.18-fold) involved in immune evasion (lactate-induced β-glucan masking) were identified.

For unique DEGs in group 2, upregulated genes (Fig. 5K) were significantly enriched in “cell wall,” “external encapsulation structure,” and “cell periphery” in CC. While downregulated genes (Fig. 5L) were significantly enriched in “ribonucleoprotein complexes,” “ribosomes,” “cytoplasm,” and “cells” in CC, “structural molecular activity” in MF, and “gene expression,” “citrate metabolism,” “tricarboxylic acid metabolism,” “organic substance metabolism,” and “purine-containing compound biosynthesis and metabolism” in BP. Only three pathways were significantly enriched in unique DEGs in group 2 (Fig. 6G), including “ribosomes,” “TCA cycle,” and “methyl butyrate metabolism.”

### Key DEGs’ alteration in *C. albicans* in 12- and 24-h interactome

Constantly up and downregulated in the whole process of interaction, 60 and 904 genes were overlapped DEGs in *C. albicans* group 1 vs 2, respectively. Eight stress response genes (*hsp90*: 4.75-fold, *ddr48*: 15.16-fold, *cdc19*: 8.35-fold, *eno1*: 3.42-fold, *gud1*: 7.80-fold, *sod4*: 7.77-fold, *tdh3*: 5.46-fold, and *ccp1*: 3.74-fold) were upregulated in mixed cultures, and *C300140WA* and *ato7* genes were potential stress response related because they were only expressed in mixed cultures. On the contrary, 29 genes were totally repressed in mixed cultures. A bunch of virulence factors showed significant downregulation, including adhesion/invasion-related genes *als1* (0.006-fold) (adhesin), *hwp2* (0.004-fold) (hyphal-associated GPI-linked protein), *mnt1* (0.20-fold) and *mnt2* (0.14-fold) (involved in O-glycosylation), phenotypic switch-related gene *efg1* (0.18-fold) (transcription factor), protease-encoding genes *sap1* (0.06-fold), and *sap9* (0.12-fold) (secreted aspartyl proteinases), biofilm formation-associated genes *bcr1* (0.22-fold) and *efg1* (0.18-fold), pH sensing-regulated genes *phr1* (0.15-fold) (contribute to cell wall assembly and morphogenesis) and *rim101* (0.22-fold) (pH response pathway), thigmotropism-associated gene *mid1* (0.20-fold) (calcium channel), stress response gene *hog1* (0.18-fold) (osmotic, oxidative, and thermal stress response), drug resistance determinants *cdr1* (0.28-fold) (transporter of the ATP-binding cassette superfamily), *erg3* (0.26-fold) (sterol desaturase), *erg11* (0.18-fold) (cytochrome P450 protein), *erg6* (0.08-fold) (methyltransferase), and *fcy2* (0.06-fold) (cytosine permease), and immune evasion-involved genes *rap1* (0.26-fold) (complement binding protein) and *ece1* (0.03-fold) (protein precursor of candidalysin) (Fig. 7C).

Concerning GO terms, upregulated DEGs (Fig. 5M) were significantly enriched in “external encapsulation structure,” “cell wall,” and “cell periphery” in CC, “pyruvate metabolism,” “monospecies metabolism,” “carboxylic acid metabolism,” and “carbohydrate (monosaccharide, hexose, and glucose) metabolism” in BP. Downregulated DEGs (Fig. 5N) were significantly enriched in “preribosome,” “ribonucleoprotein complex,” and “intrinsic components of membrane” in CC and “carboxylic acid activity,” “ion activity,” and “transmembrane transporter activity” in MF. In overlapped DEGs in group 1 vs 2, seven pathways were significantly enriched (Fig. 6H), including “Ribosome biogenesis in eukaryote,” “RNA polymerase,” “purine metabolism,” “pyrimidine metabolism,” “taurine and hypotaurine metabolism,” “glycerophospholipid metabolism,” and “mitogen-activated protein kinase (MAPK) signaling pathway.”

### Key DEGs during maturation of *L. plantarum* from 12 to 24 h

Representing the key genes contributing to the reduced proliferation rate of *C. albicans* yeast cells in mixed culture, 2 up- and 98 downregulated (seven had no expression in sample LhCa_24 h) DEGs were identified uniquely in *C. albicans* group 4. Regarding GO terms, downregulated DEGs (Fig. 5O) were significantly enriched in “ribonucleoprotein complex,” “polymer complex,” “ribosome,” and “cytoplasm” in CC, “structural molecular activity” in MF, and “gene expression,” “citric acid metabolism,” and “tricarboxylic acid metabolism” in BP. A total of five pathways were significantly enriched in unique DEGs in group 4 (Fig. 6I), including “ribosomes,” “TCA cycle,” “oxidative phosphorylation,” “carbon metabolism,” and “glyoxylic acid and dicarboxylic acid metabolism.” In addition, the enriched pathways were compared among the four groups (Fig. 6J).

## DISCUSSION

Studies on human genital and gastrointestinal tract microbiota have revealed *Lactobacillus* spp. as a frequently identified, even dominant bacterial genus (29, 41–43), mostly associated with health benefits, and *C. albicans* as an opportunistic fungal pathogen inducing candidiasis (12). *L. plantarum* strains from diverse niches, including the human oral cavity, vagina, feces, and fermented foods, had been tested to acquire anti-*C*. *albicans* activity and were considered a promising probiotic treatment for VVC (30–34). However, the anti-*C*. *albicans* activity differs among *Lactobacillus* species and strains and is influenced by various factors (19–22). In addition, most studies have focused on the anti-hyphal formation effect, eliminating the importance of *C. albicans* yeast form, the proliferation of hyphae cells, and the effect of *C. albicans* posed on *L. plantarum*. In this study, a mixed culture model of *L. plantarum* BM-LP14723 and *C. albicans* SC5314 yeast and hyphae cells, respectively, was setup, and the changes in proliferation, morphology, and transcriptomes of both strains were monitored.

In the mixed culture of *L. plantarum* and *C. albicans* yeast cells, yeast cell proliferation was suspended when *L. plantarum* entered the stationary phase, while the growth of *L. plantarum* was stable and uninfluenced. In addition, the inhibition of *L. plantarum* on *C. albicans* yeast cell proliferation depended on *L. plantarum* cellular density. The inhibition effect was somewhat consistent with previous studies showing that *L. plantarum* strains acquired anti-*C*. *albicans* activity (30–34). However, no change was observed in proliferation level in the mixed culture of *L. plantarum* and *C. albicans* hyphal cells. As a polymorphic fungus, specific environmental cues can trigger *C. albicans* to undergo morphological transition from yeast to hyphae (25). Hyphal cells are known to be more robust and acquire stronger resistance to stress, which might contribute to their unchanged proliferation level in the mixed culture with *L. plantarum*. Considering *C. albicans* stays as yeast cells in the oral cavity, intestinal tract, and genital tract of healthy human bodies where they co-exist with *L. plantarum*, it is important to explore their interaction and how they stay (13, 14). In a subsequent investigation, we emphasized the interaction between *L. plantarum* and *C. albicans* yeast cells.

Short-term co-aggregation through physical interaction had previously been revealed in the early stage of *L. plantarum* and *C. albicans* interaction (44). To examine the cell-to-cell interaction between *L. plantarum* and *C. albicans* yeast cells, optical microscopy and AFM were applied to observe the morphological changes. Interestingly, co-aggregation with *L. plantarum* cells surrounding *C. albicans* yeast cells was recorded, demonstrating direct cell-cell contact and interaction. The co-aggregation rate between *L. plantarum* 319 and *C. albicans* cells had been reported to be the highest among five strains of intestinal Lactobacilli and seven clinical isolates of *Candida* (44). Among the five strains, *Lacticaseibacillus rhamnosus* IMC 501, *Lacticaseibacillus paracasei* IMC 502, and SYNBIO were able to produce H_2_O_2_ to co-aggregate and exert antimicrobial activity against pathogenic *Candida* strains (44). Biosurfactants and bacteriocins secreted by Lactobacilli have also been suggested to contribute to its increased adhesion (45, 46).

Besides biosurfactants and bacteriocins, H_2_O_2_, lactic acid, exopolysaccharides, and fatty acids produced by Lactobacilli have been key factors contributing to the inhibition of *C. albicans* growth (9, 18, 19). In the current study, we also tested the role of *L. plantarum* CFCS in reduced *C. albicans* yeast cell proliferation. CFCS from mature *L. plantarum* culture (>24 h) and CFCS from *L. plantarum-C. albicans* mixed culture to a higher level contributed to the reduced *C. albicans* yeast cell proliferation. However, the inhibition rates were lower than *L. plantarum* culture, suggesting the important role of *L. plantarum* cells in the interaction. After co-existence for 24 h, the pH of Lactobacilli-*C. albicans* mixed culture (3.7–4.2) had been identified to be significantly lower than *C. albicans* single culture (5.3–5.8) (47). Thus, the decreased *C. albicans* cell number in mixed culture has been suggested due to the lactic acid produced during Lactobacilli metabolism (47). However, the lactic acid produced by *Lacticaseibacillus rhamnosus* GG has been shown to have no effect on *C. albicans* growth (19). In this study, the pH values of CFCS from *L. plantarum* single culture and *L. plantarum-C. albicans* culture were determined, and pH-adjusted media were used to examine the influence of low pH on *C. albicans* proliferation. The results showed that CFCS mimicking acidified medium was not sufficient to cause reduced *C. albicans* yeast cell proliferation. The acidic environment created by *L. plantarum* could not inhibit *C. albicans* growth.

Phenotypically, in *L. plantarum-C. albicans* mixed culture, the proliferation of *L. plantarum* remained unchanged and that of *C. albicans* yeast cell was inhibited after 12 h. But it did not mean no change was made by *L. plantarum* cells. Intrinsic changes should have happened in both *L. plantarum* and *C. albicans* cells, leading to the reduced *C. albicans* yeast cell proliferation, co-aggregation, and higher-level influence of CFCS from mixed culture on *C. albicans* yeast cells. To explore such changes, regular RNA-seq was performed on *C. albicans* single culture and *L. plantarum* single culture, and polymicrobial RNA-seq was performed on *L. plantarum-C. albicans* mixed culture. Two time points were selected, with 12 h as unchanged cell proliferation on both cells and 24 h as reduced *C. albicans* cell proliferation. Downstream key DEGs’ identification and GO term and KEGG pathway enrichment were performed to acquire the gene regulation and pathway changes. In previous studies, genetic changes in *C. albicans,* especially the expression level of selected virulence factors, had been studied by reverse transcription-polymerase chain reaction (RT-PCR). Considering the limitation of RT-PCR, which could identify single gene expression and complication of genetic polymorphism, global transcriptomics analyses by RNA-seq and bioinformatics were conducted in this study. Importantly, the global changes in both *L. plantarum* and *C. albicans* were analyzed, with a few interesting points elucidated.

First, two *L. plantarum* QS systems (*lamBDCA* and *luxS*) showed significant changes in mixed culture. Two types of QS systems, including two-component signaling system and *luxS*/AI-2 signaling system, had been identified in *L. plantarum*. Concerning two-component signaling system, *lamBDCA* and *lamKR* systems have been found to be cooperative in *L. plantarum* WCFS1, homologous to the *agrBDCA* system in *Staphylococcus aureus* and the *fsrABC* system in *Enterococcus faecalis* (40, 48). The transcriptional regulation of the *lam* QS system relies on growth status. In the single culture of *L. plantarum*, the genes in the lam QS system were found to start expression at 5 h and maintain continuous high expression during post-logarithmic phase and stationary phase (48). In this study, the genes in the *lamBDCA* system showed significant upregulation in *L. plantarum-C. albicans* mixed culture, indicating the existence of *C. albicans* yeast cells induces the activation of the *lamBDCA* system. The mutation of the *lamA* gene in the *lamBDCA* system had been linked to reduced adhesion activity (48), suggesting the possible contribution of the upregulated *lamBDCA* system to the co-aggregation of *L. plantarum* and *C. albicans* cells. Similarly, in *S. aureus-C. albicans* mixed culture, the *lamBDCA* homologous *agrBDCA* system in *S. aureus* had been revealed to be induced by *C. albicans* (49, 50). Such “coincidence” may indicate that there is a possible link in such interactions. Another QS system, *luxS*/AI-2, is a common system in many Gram-negative and Gram-positive bacteria, contributing to interspecific communication (51). In Lactobacilli, the *luxS* gene had been shown to be significantly upregulated in the interaction with *Escherichia coli* O157:H7, *Listeria monocytogenes,* and *S. aureus* (52, 53). However, in the current study, the *L. plantarum luxS* gene was significantly downregulated in mixed culture with *C. albicans*. Genes involved in the AI-2 production pathway, including *metE*, *metH*, and *metK* (54), were also downregulated in mixed culture, indicating the inactivation of the *luxS*/AI-2 system in *L. plantarum* by *C. albicans*. A large scale of genes previously identified to be upregulated by AI-2 in *Lactiplantibacillus paraplantarum* L-ZS9 (54) were also mostly downregulated in this study. The discrepancy elucidated the difference between *L. plantarum* and *C. albicans* in communication with other bacteria and suggested the diversity in bacteria-bacteria communication and bacteria-fungi communication. The difference was potentially because the QS systems carried by other bacteria are unique and not identified in fungi (55). Especially the acyl homoserine lactone QS system carried in Gram-negative strains might play a role in inducing the activation of the luxS/AI-2 system in *L. plantarum*. Besides the signaling factors involved in QS systems, some other signaling factors also showed significant change, mostly upregulation, which are potential stimulating factors for *C. albicans*.

**Fig 8:**
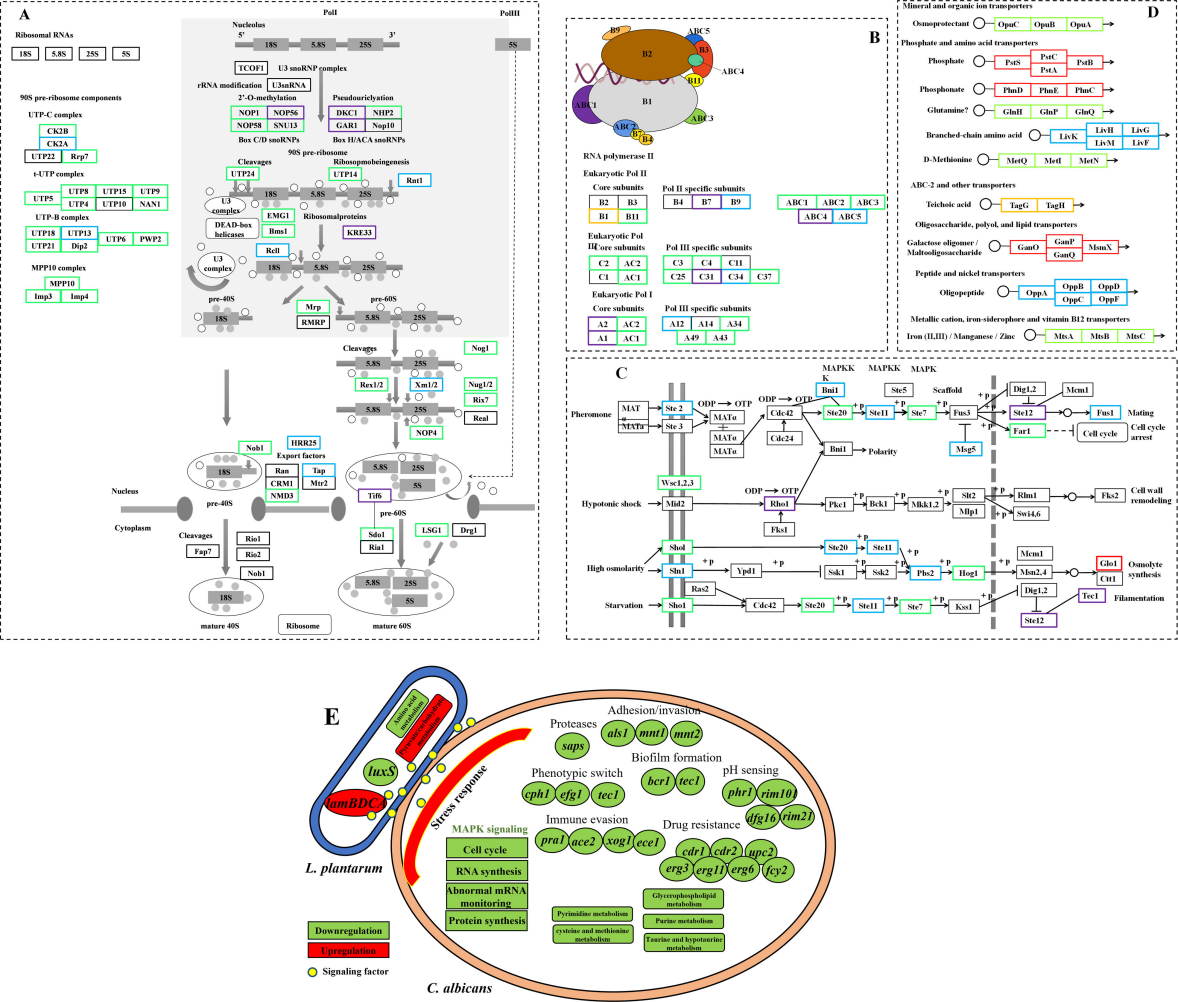
Gene expression changes in ribosome neogenesis (**A**), RNA polymerase (**B**), MAPK signaling pathway (**C**), pathways from *C. albicans,* and ABC transporter pathway (**D**) from *L. plantarum*, as well as the dual-species interaction between *L. plantarum* and *C. albicans* (**E**). Red: both upregulated in groups 1 and 2; green: both downregulated in groups 1 and 2; blue: downregulated in groups 1; purple: downregulated in group 2; and yellow: upregulated in group 2.

Second, in mixed culture, the upregulation of stress response-related genes and downregulation of cell cycle, cell survival, and cell integrity-related pathways were identified in *C. albicans*, possibly connected to the stress posed by *L. plantarum* and the reduced yeast cell proliferation. The upregulated stress response-related genes were involved in response to oxidative stress, external chemical stimuli, acidic pH, nutritional environment, organic substances, and metal ions. Simultaneously, *C. albicans* downregulated ribosomal neogenesis (Fig. 8A), spliceosomes, RNA polymerase (Fig. 8B), matrix transcription factors, cell cycle, meiosis, and MAPK signaling pathway (Fig. 8C) in the early stage, and downregulated ribosomes, ribosome neogenesis, RNA polymerase, and MAPK signaling pathways in the later stage of mixed culture. Ribosome neogenesis includes the synthesis and precise assembly of four rRNAs and approximately 80 ribosomal proteins (56). The downregulation of the ribosome neogenesis pathway might lead to the termination of microbial growth. The cell cycle is a process of repeated DNA replication and mitosis and its intermediate stages (57). The downregulation of the cell cycle pathway indicated that *C. albicans* slowed down the progress of the cell cycle in mixed culture, potentially resulting in slower proliferation. The downregulation of RNA polymerase and matrix transcription factor pathways indicated a decrease in *C. albicans* RNA synthesis. The downregulation of the mRNA monitoring pathway, which is a quality control mechanism for detecting and degrading abnormal mRNA, reduced the ability of *C. albicans* to monitor abnormal mRNA, which might have a greater impact on the growth and metabolism due to the presence of abnormal mRNA. The transition between growth and meiosis in yeast is regulated by nutrient signals, and the downregulation of meiotic pathways indicates that external nutrient signals regulate the conversion of its focus of gravity to growth (58). Therefore, it is possible that *C. albicans* downregulated cell cycle, RNA synthesis, abnormal mRNA monitoring, and protein synthesis in the early stage and RNA and protein synthesis in the later stage of mixed culture, resulting in a decreased proliferation rate. The partial MAPK signaling pathway [especially the high-osmolarity glycerol pathway contributing to adaption to stress, morphogenesis, and cell wall formation (59)] (Fig. 8C) was also downregulated in the mixed culture, potentially contributing to the reduced proliferation rate of *C. albicans*.

Third, a large scale of pathogenesis and virulence factors were downregulated in *C. albicans* (Fig. 5C), including adhesion/invasion (*als1*, *mnt1*, and *mnt2*), phenotypic switch (*cph1*, *efg1*, and *tec1*), proteases (*sap1*, *sap2*, *sap5*, *sap6*, *sap8*, *sap9*, and *sap10*), nutrient acquisition (*pra1*), environmental adaption and biofilm formation (*bcr1*, *tec1*, and *efg1*), pH sensing (*phr1*, *rim101*, *dfg16*, and *rim21*), thigmotropism (*mid1*), drug resistance (*cdr1*, *cdr2*, *upc2*, *erg3*, *erg11*, *erg6*, and *fcy2*), and immune evasion (*pra1*, *ace2*, *xog1*, and *ece1*)-associated genes. The adhesion and invasion ability enable *C. albicans* to adhere and penetrate into host cells (60). The downregulation of adhesion/invasion-related genes in mixed culture indicated the potential interruption of *L. plantarum* to *C. albicans* adherence/invasion ability. However, the expression of *als3* and *hwp1* (61, 62), which are major adhesin and invasion in *C. albicans,* remained stable in mixed culture, indicating the limited influence of *L. plantarum* on *C. albicans* adhesion/invasion. The downregulation of *als3* and *hwp1*, as well as transcriptional regulator *bcr1* and *cph1,* had been previously identified in *C. albicans* co-cultured with *Lacticaseibacillus rhamnosus* (63). In *C. albicans* pathogenesis, adhesion to host cells is followed by hyphae formation, which is accompanied by the expression of hypha-associated proteins with known damage and immune activation capabilities (64–66). *Cph1* and *efg1* deletion mutants had been well documented to lose the ability to form hyphae. In the mixed culture of *L. plantarum* and *C. albicans*, hyphae formation-associated genes, *cph1*, *efg1,* and *tec1,* were significantly downregulated, indicating the reduced yeast-hyphae transition ability of *C. albicans* in the presence of *L. plantarum*. Secretion of proteases facilitates active penetration of hyphae into epithelia contributing to extracellular nutrient acquisition (67). The downregulation of some secreted aspartic proteinases (Saps) and nutrient acquisition protein-encoding genes in mixed culture revealed the compromised penetration activity of *C. albicans*. Importantly, the *ece1* gene, which encodes a polypeptide generating candidalysin, showed significant downregulation (32-fold) in mixed culture. Candidalysin is a newly discovered toxin contributing to the pathogenesis of *C. albicans* in multiple infection models (68–70). The repression of the *ece1* gene by *L. plantarum* indicated that it significantly impacts the pathogenesis of *C. albicans*.

Fourth, partial metabolism and transport pathways were changed in *L. plantarum* and *C. albicans* in mixed culture. In *L. plantarum*, significantly changed pathways included upregulation of pyruvate and carbohydrate metabolism and downregulation of amino acid (histidine, glycine, serine, threonine, phenylalanine, tyrosine, tryptophan, cysteine, and methionine) metabolism pathways in early stage and upregulation of pyruvate metabolism pathway and downregulation of amino acid (phenylalanine, tyrosine, and tryptophan) metabolism pathways in the later stage. During the whole process, multiple ABC transporters were consistently up/downregulated in *L. plantarum* (Fig. 8D). In *C. albicans*, the downregulation of the glycerophospholipid metabolism pathway in the early stage and purine metabolism, taurine and hypotaurine metabolism, pyrimidine metabolism, and cysteine and methionine metabolism pathways in the later stage was identified.

### Conclusion

In this study, taking both yeast and hyphal forms of *C. albicans* into consideration, we set up a dual-species interaction model of *L. plantarum* and *C. albicans* and investigated their changes in proliferation, morphology, and transcriptomes (Fig. 8E). Maintaining stable and unchanged growth rate, *L. plantarum* inhibited *C. albicans* yeast cell proliferation but failed to induce reduced hyphal growth. The inhibition of *L. plantarum* on *C. albicans* yeast cell proliferation was dependent on *L. plantarum* cell density. Combining optical microscopy and AFM, cell-to-cell direct contact and co-aggregation with *L. plantarum* cells surrounding *C. albicans* yeast cells were observed during dual-species interaction. Reduced *C. albicans* yeast cell proliferation in mixed culture was partially due to *L. plantarum* CFCS but not acidic environment. Upon polymicrobial transcriptomics analysis, interesting changes were identified in both *L. plantarum* and *C. albicans* gene expression. First, two *L. plantarum* QS systems showed contrary changes, with the activation of *lamBDCA* and repression of *luxS*. Second, the upregulation of stress response-related genes and downregulation of cell cycle, cell survival, and cell integrity-related pathways were identified in *C. albicans*, possibly connected to the stress posed by *L. plantarum* and the reduced yeast cell proliferation. Third, a large scale of pathogenesis and virulence factors were downregulated in *C. albicans*, indicating the potential interruption of pathogenic activities by *L. plantarum*. Fourth, partial metabolism and transport pathways were changed in *L. plantarum* and *C. albicans*. The information yield in this study might aid in understanding the behavior of *L. plantarum* and *C. albicans* in dual-species interaction.

## MATERIALS AND METHODS

### Strains and growth conditions

*C. albicans* strain SC5314 and *L. plantarum* strain BM-LP14723 were maintained as glycerol stock stored at −80°C. A small amount of glycerol stock was spread onto appropriate agar and incubated at optimum conditions (37°C for 48 h for *C. albicans* and 30°C for 24 h for *L. plantarum*) to obtain single colonies. A single colony was transferred to 2 mL of appropriate broth and incubated at optimum temperature with shaking at 200 rpm overnight prior to further experiments. MRS medium was used for the optimal growth of *L. plantarum*. For *C. albicans*, YPD and Roswell Park Memorial Institute-1640 medium were used for the growth of yeast and hyphal cells, respectively.

### Initial assessment by modified agar overlay assay

Agar overlay assay was conducted to assess the inhibition of *L. plantarum* on *C. albicans* growth according to previous studies with slight modification (23, 24). Overnight culture of *L. plantarum* was spotted/streaked onto the MRS agar plate and incubated at 37°C for 24 h to develop visible microcolony/culture line. Thereafter, the overnight culture of *C. albicans* was adjusted to a predetermined optical density (approximately 10^7^ CFU/mL) to yield confluent growth in 12 mL of 0.7% YPD agar (top agar). The top agar (melted and cooled to 42°C) was seeded with the overnight culture of *C. albicans* and poured over the microcolony/culture line of *L. plantarum*. The plate was incubated for 24 h at 37°C to yield inhibitory zones.

### Growth of *L. plantarum* and *C. albicans* in mixed culture

A mixed culture consisting of *C. albicans* and *L. plantarum* cells at a ratio of 1:100, 1:1, and 100:1, respectively, was set up and incubated at 37°C with shaking at 200 rpm. Single cultures of *C. albicans* and *L. plantarum* served as controls. During incubation, 10 µL of mixed/single culture was retracted every 4 h and dropped on MRS agar supplemented with 10 µg/mL of amphotericin B (Shanghai Yuanye Bio-Technology Co., Ltd, China) and YPD agar supplemented with 200 µg/mL of chloramphenicol (Shanghai Yuanye Bio-Technology Co., Ltd, China) for selective growth of *L. plantarum* and *C. albicans*, respectively.

### Morphology observation of *L. plantarum* and *C. albicans* in mixed culture

Specifically, *L. plantarum* and *C. albicans* cells from 12- and 24-h single and mixed cultures were collected and observed under Axioskop 40 Pol optical microscope (Zeiss, Germany) and AFM XE-100 (Park Systems, USA). Upon observation under AFM, cell samples were prepared following the instructions in a previous publication (71). Data were recorded for at least 10 fields of view per sample, and results represent typical observation in each field. The recorded observation was adapted to surface roughness determination using XEI software.

### *L. plantarum* CFCS preparation

Phosphate-buffered saline (PBS)-washed cells of *L. plantarum* from overnight culture were resuspended in MRS broth with a final concentration of 10^7^ or 10^5^ CFU/mL and incubated at 37°C with shaking at 200 rpm for 6, 12, 24, and 48 h, respectively. *L. plantarum* culture was collected and centrifuged at 5,000 rpm for 1 min. Supernatants were pipetted out and filtered through a 0.22 µm microfilter (Millipore, USA). The CFCS collected from 6-, 12-, 24-, and 48-h *L*. *plantarum* cultures were designated 6 h Lacto S, 12 h Lacto S, 24 h Lacto S, and 48 h Lacto S, respectively.

### CFCS preparation from mixed culture

The mixed culture of *C. albicans* and *L. plantarum* cells at a ratio of 1:100, 1:1, and 100:1 was setup as mentioned above and incubated for 6, 12, 24, and 48 h, respectively. Mixed cultures were collected and centrifuged at 5,000 rpm for 1 min. Supernatants were pipetted out and filtered through a 0.22 µm microfilter (Millipore, USA). The CFCS collected from 6-, 12-, 24-, and 48-h mixed cultures were designated 6 h (Lacto + Can) S, 12 h (Lacto + Can) S, 24 h (Lacto + Can) S, and 48 h (Lacto + Can) S, respectively.

### Growth of *C. albicans* with the supplement of CFCS

PBS-washed cells of *C. albicans* from the overnight culture were resuspended in YPD broth with a final concentration of 10^7^ or 10^5^ CFU/mL. *C. albicans* culture was mixed in the ratio of 1:1 with CFCS collected from single or mixed cultures. For the groups with single culture CFCS, *C. albicans* culture mixed in the ratio of 1:1 with MRS broth or distilled H_2_O served as a control. For the groups with mixed culture CFCS, *C. albicans* culture mixed in the ratio of 1:1 with MRS-YPD broth (1:1 mixture of MRS and YPD broth) or distilled H_2_O was used as control. All experimental groups were incubated at 37°C with shaking at 200 rpm. Optical density value at 600 nm (OD_600_) was determined every 2 h using an InfiniteM200 Pro multimode plate reader (Tecan, Switzerland), and CFU counting on selective YPD agar was performed at 12 and 24 h.

### Growth of *C. albicans* in acidified medium

The pH values of 12 h Lacto S, 24 h Lacto S, 12 h (Lacto + Can) S, and 24 h (Lacto + Can) S were measured using a PHS-3C pH indicator (Shanghai Puchun Measure Instrument Co., Ltd., China). The pH of MRS broth was adjusted to be equivalent to that of 12 h Lacto S and 24 h Lacto S, respectively. The pH of MRS-YPD was adjusted to be equivalent to that of 12 h (Lacto + Can) S and 24 h (Lacto +Can) S, respectively. *C. albicans* culture was mixed in the ratio of 1:1 with pH-adjusted MRS or MRS-YPD, with corresponding CFCS as controls. All experimental groups were incubated at 37°C with shaking at 200 rpm. OD_600_ was measured every 2 h using InfiniteM200 Pro multimode plate reader (Tecan, Switzerland).

### Polymicrobial library construction and RNA-seq

Mixed cultures of *C. albicans* yeast cells and *L. plantarum* cells, together with respective single cultures, were collected in biological triplicate at specific time points. The cells were collected by centrifugation and adapted to fast freezing in liquid nitrogen. Total RNA was extracted using TRizol reagent (Sigma-Aldrich, USA) based on the manufacturer’s instructions. Considering the difference between *C. albicans* and *L. plantarum* cells, eukaryotic mRNA from *C. albicans* was enriched by Oligo(dT) beads, while prokaryotic mRNA from *L. plantarum* was enriched by removing rRNA by Ribo Zero Magnetic Kit (Epicentre). mRNA from mixed cells was adapted to both enrichment processes. A bacterial sequencing library was constructed for *L. plantarum* single culture groups, followed by sequencing through the Illumina HiSeq 2500 platform with a depth of 1 Gb. A fungal sequencing library was constructed for *C. albicans* single culture groups, followed by sequencing through the Illumina HiSeq 2500 platform with a depth of 3 Gb. A polymicrobial sequencing library was constructed for mixed culture groups, followed by sequencing through Illumina HiSeq 2500 platform with a depth of 3.5 Gb. The library construction and RNA-seq were performed by Gene Denovo Biotechnology Co. (Guangzhou, China). Raw reads were adapted to filtration to get high-quality clean reads, which were further quality examined by FastQC v.0.10.1 (http://www.bioinformatics.babraham.ac.uk/projects/fastqc/). The clean reads were assembled and aligned to the reference genome using TopHat2 (version 2.0.3.12) (72). The genomes of *C. albicans* strain SC5314 and *L. plantarum* strain WCFS1 were used as reference genomes.

### DEGs’ identification and functional enrichment

The gene expression level was normalized by the FPKM method. To identify DEGs in a comparative group, the edge R package (http://www.r-project.org/) was used. DEGs with log_2_ǀfold-changeǀ ≥ 1 and false discovery rate < 0.05 were considered to be significant. DEGs were annotated against the Nr database, GO database (73, 74), and KEGG pathway database (73, 74). GO terms and KEGG pathway enrichment analyses were performed to identify key DEGs and pathways.

### Statistical analysis

The experimental data were shown as the mean ± standard deviation from at least three replicates. Statistical differences among the results obtained by the treatments and control were examined by Student *t*-test and one-way analysis of variance (75), followed by Tukey’s multiple intergroup comparison, where appropriate. Fisher’s exact test was used in KEGG pathway enrichment analysis, and hypergeometric distribution was used to identify significantly enriched GO terms. A *P* value < 0.05 was considered statistically significant.

## Data Availability

The RNA-seq data from the mixed culture of *C. albicans* yeast cells and *L. plantarum* cells, together with respective single culture, were deposited in the NCBI SRA database under BioProject accession number PRJNA901698.

## SUPPLEMENTAL MATERIAL

Supplemental figures spectrum.00353-24-s0001.docx Fig. S1-S3.

Table S1 spectrum.00353-24-s0002.docx Statistics of differentially expressed gene numbers.

Table S2 spectrum.00353-24-s0003.xlsx DEGs in each comparative group.

Table S3 spectrum.00353-24-s0004.xlsx Unique and overlap DEGs between comparative groups.

ASM does not own the copyrights to Supplemental Material that may be linked to, or accessed through, an article. The authors have granted ASM a non-exclusive, world-wide license to publish the Supplemental Material files. Please contact the corresponding author directly for reuse.
