# Supplementary material for: Differential alteration in Lactiplantibacillus plantarum subsp. plantarum quorum-sensing systems and reduced Candida albicans yeast survival and virulence gene expression in dual-species interaction
Source: Microbiol Spectr. 2024 May 8;12(6):e00353-24. doi: 10.1128/spectrum.00353-24 (PMC11237386; doi:10.1128/spectrum.00353-24)
Supplement: Supplemental figures — Fig. S1-S3. [file spectrum.00353-24-s0001.docx]

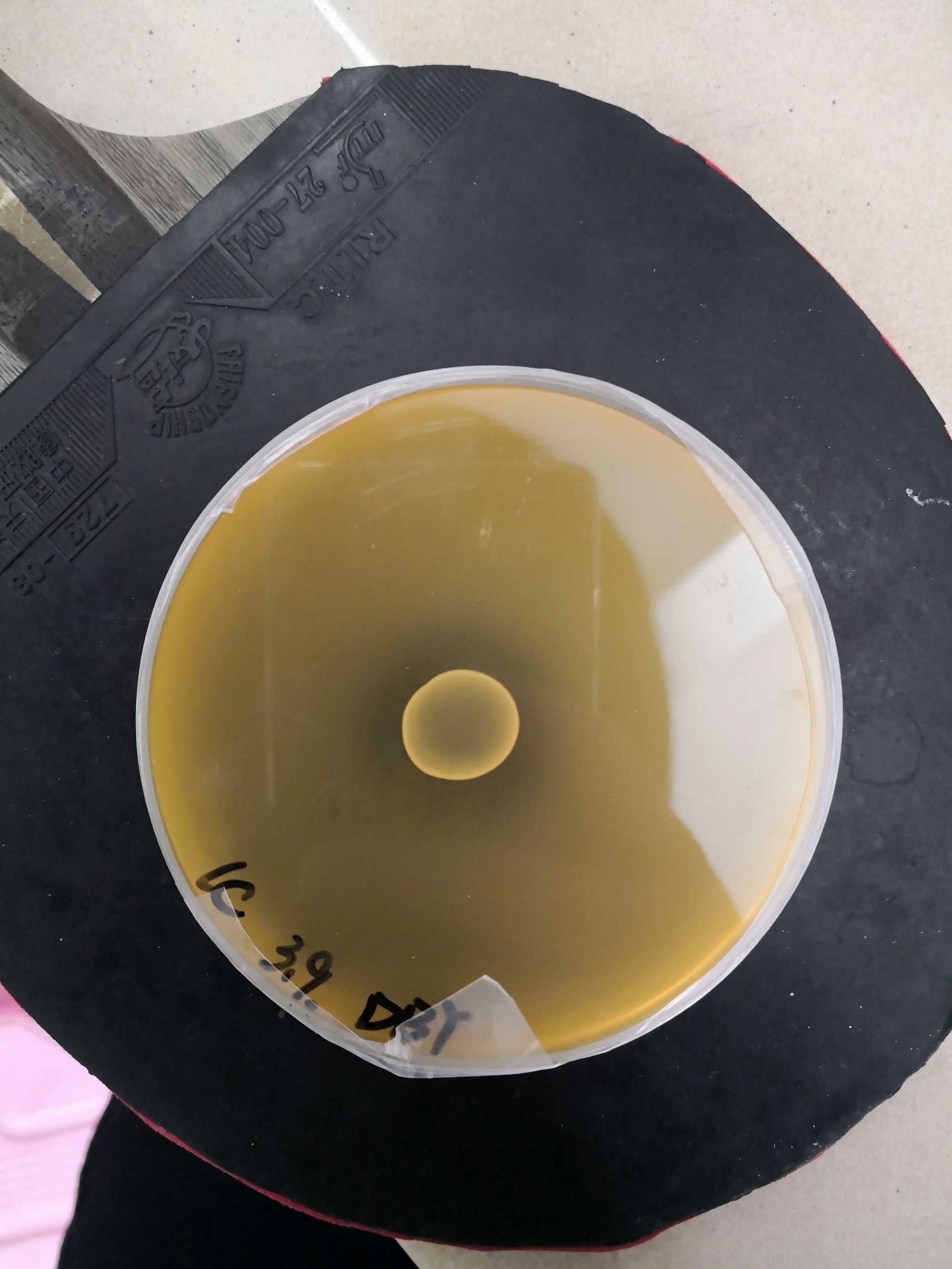

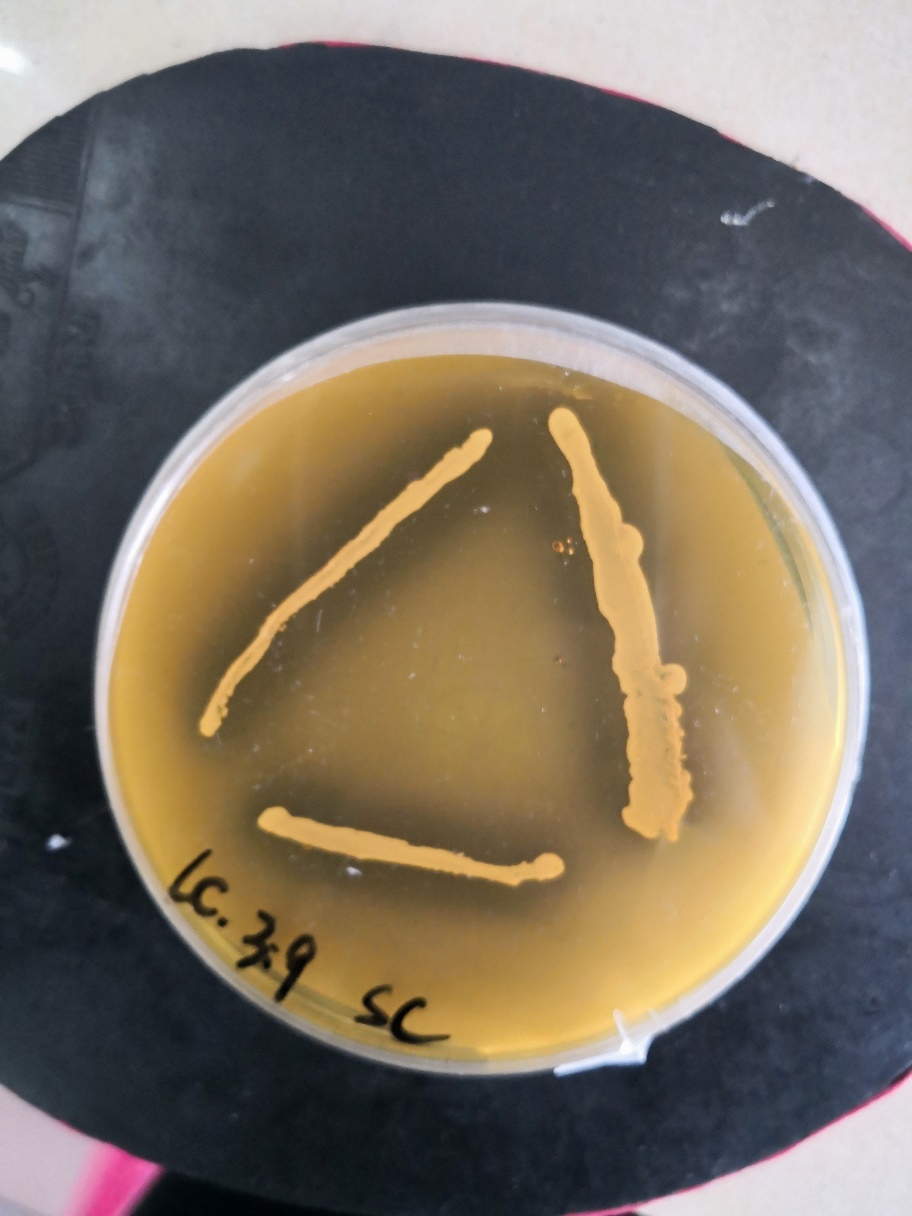


**A B**

Figure S1. Inhibitory zones (circled by red) around spotted microcolony (A) or streaked culture line (B) of *L. plantarum* to *C. albicans* (mixed with agar).

Figure S2. Yeast cell proliferation in groups supplemented with CFCS from *L. plantarum* single culture examined by OD_600_ (A, B) and CFU (C, D), with different initial concentrations. A&C: 10^7^ Lacto S+10^5^ Can (10^5^ CFU/mL *C. albicans* yeast cells were mixed with supernatant collected from 10^7^ CFU/mL *L. plantarum* single culture for 6 h, 12 h, 24 h, and 48 h, respectively), B&D: 10^5^ Lacto S+10^7^ Can (10^7^ CFU/mL *C. albicans* yeast cells were mixed with supernatant collected from 10^5^ CFU/mL *L. plantarum* single culture for 6 h, 12 h, 24 h, and 48 h, respectively).

Figure S3. Yeast cell proliferation in groups supplemented with CFCS from mixed culture examined by OD_600_ (A-E) and CFU (F-K). A&F: (10^7^ Lacto+10^7^ Can) S+10^5^ Can (10^5^ CFU/mL *C. albicans* yeast cells were mixed with supernatant collected from mixed culture of 10^7^ CFU/mL *L. plantarum* and 10^7^ CFU/mL *C. albicans* yeast cells for 6 h, 12 h, 24 h, and 48 h, respectively *S. cerevisiae*), B&G: (10^7^ Lacto+10^5^ Can) S+10^7^ Can (10^7^ CFU/mL *C. albicans* yeast cells were mixed with supernatant collected from mixed culture of 10^7^ CFU/mL *L. plantarum* and 10^5^ CFU/mL *C. albicans* yeast cells for 6 h, 12 h, 24 h, and 48 h, respectively *S. cerevisiae*), C&H: (10^7^ Lacto+10^5^ Can) S+10^5^ Can (10^5^ CFU/mL *C. albicans* yeast cells were mixed with supernatant collected from mixed culture of 10^7^ CFU/mL *L. plantarum* and 10^5^ CFU/mL *C. albicans* yeast cells for 6 h, 12 h, 24 h, and 48 h, respectively *S. cerevisiae*), D&I: (10^5^ Lacto+10^7^ Can) S+10^7^ Can (10^7^ CFU/mL *C. albicans* yeast cells were mixed with supernatant collected from mixed culture of 10^5^ CFU/mL *L. plantarum* and 10^7^ CFU/mL *C. albicans* yeast cells for 6 h, 12 h, 24 h, and 48 h, respectively *S. cerevisiae*), E&J: (10^5^ Lacto+10^7^ Can) S+10^5^ Can (10^5^ CFU/mL *C. albicans* yeast cells were mixed with supernatant collected from mixed culture of 10^5^ CFU/mL *L. plantarum* and 10^7^ CFU/mL *C. albicans* yeast cells for 6 h, 12 h, 24 h, and 48 h, respectively *S. cerevisiae*). K: Comparison among groups with different initial concentrations.
