# Supplementary material for: Differential alteration in Lactiplantibacillus plantarum subsp. plantarum quorum-sensing systems and reduced Candida albicans yeast survival and virulence gene expression in dual-species interaction
Source: Microbiol Spectr. 2024 May 8;12(6):e00353-24. doi: 10.1128/spectrum.00353-24 (PMC11237386; doi:10.1128/spectrum.00353-24)
Supplement: Table S1 — Statistics of differentially expressed gene numbers. [file spectrum.00353-24-s0002.docx]

Table S1 Statistics of differentially expressed gene numbers

| **Comparative groups** | **Up regulated gene** | **Down regulated gene** | **Differentially expressed gene** |
| --- | --- | --- | --- |
| Lh_12h vs Lh_24h | 181 | 537 | 718 |
| Lh_12h vs LhCa_12h | 234 | 960 | 1194 |
| Lh_24h vs LhCa_24h | 477 | 658 | 1135 |
| LhCa_12h vs LhCa_24h | 145 | 89 | 234 |
| Ca_12h vs Ca_24h | 16 | 26 | 42 |
| Ca_12h vs LhCa_12h | 140 | 2310 | 2450 |
| Ca_24h vs LhCa_24h | 130 | 1252 | 1382 |
| LhCa_12h vs LhCa_24h | 2 | 98 | 100 |
